# Entropy plateaus, combinatorial degeneracy, local moments, and heavy-fermion mass renormalization in magic-angle graphene

**DOI:** 10.1038/s41467-026-76179-y

**Published:** 2026-08-08

**Authors:** Zhenyuan Zhang, Shuang Wu, Dumitru Călugăru, Haoyu Hu, Takashi Taniguchi, Kenji Wanatabe, Andrei B. Bernevig, Eva Y. Andrei

**Affiliations:** 1 Department of Physics and Astronomy, Rutgers University, Piscataway, New Jersey 08854 USA; 2 Department of Physics, Princeton University, Princeton, New Jersey 08544 USA; 3 Rudolf Peierls Centre for Theoretical Physics, University of Oxford, Oxford, OX1 3PU United Kingdom; 4 Donostia International Physics Center (DIPC), Paseo Manuel de Lardizabal, 20018 San Sebastián, Spain; 5 International Center for Materials Nanoarchitectonics, National Institute for Materials Science, 1- 1 Namiki, Tsukuba, 305-0044 Japan; 6 Research Center for Functional Materials, National Institute for Materials Science, 1-1 Namiki, Tsukuba, 305-0044 Japan; 7 IKERBASQUE, Basque Foundation for Science, 48013 Bilbao, Spain

**Keywords:** Electronic properties and materials, Graphene, Phase transitions and critical phenomena

## Abstract

Heavy-fermion behavior, driven by the interaction-induced enhancement of electronic mass, underpins exotic states ranging from unconventional superconductivity to quantum criticality. Long restricted to complex three-dimensional *f-*electron compounds, these phenomena are now predicted to emerge within the flat bands of magic-angle twisted bilayer graphene. Here, we use high-resolution planar tunneling spectroscopy to perform inverse-compressibility and entropy measurements, providing direct evidence for topological heavy-fermion behavior in magic-angle graphene. Our inverse-compressibility data reveal strong, filling-dependent mass renormalization, consistent with the hybridization between localized and itinerant electrons within a Kondo-lattice framework. Furthermore, entropy spectroscopy reveals two plateau structures whose degeneracies directly encode the combinatorial structure of local moment states. This 8-to-4-fold degeneracy reduction is an  entropic signature of strain-mediated symmetry breaking, consistent with the topological heavy-fermion model.

## Abstract (web summary)

Magic-angle graphene is known to host flat bands and emergent phenomena. Here, the authors provide thermodynamic evidence for topological heavy-fermion behavior, observing a strain-induced reduction in the combinatorial degeneracy of isospin local moments (from eight-fold to four-fold) alongside pronounced electronic mass renormalization near integer band fillings.

## Introduction

Heavy-fermion behavior, marked by strong interaction–driven enhancements of the electronic effective mass, underlies a range of exotic quantum phenomena, including unconventional superconductivity and quantum criticality^1^. Although traditionally realized in chemically complex, three-dimensional *f*-electron compounds^2^, such behavior has recently been predicted to emerge in the flat bands of magic-angle twisted bilayer graphene (MATBG), a clean and highly tunable two-dimensional system^3–6^. Twisted bilayer graphene develops a moiré superlattice potential that reconstructs the electronic band structure. Near the magic twist angle ( ~ 1.1°)^7–9^ MATBG hosts two 4-fold degenerate flat bands that are isolated from the high-energy remote bands. The flat bands dramatically suppress the electron kinetic energy, increasing their effective mass and enhancing the role of electron-electron interactions, paralleling traditional heavy fermion materials. As a result, emergent phenomena can arise, including unconventional superconductivity^10,11^, non-Fermi liquid behavior^12,13^, topologically nontrivial phases^14–17^, and band reconstruction^7,9^ manifested as a cascade of electronic transitions at integer moiré fillings^17–19^. These observations motivated topological heavy fermion (THF) models^3–6,20^ describing the flat bands in MATBG as arising from the hybridization between localized flat-band orbitals (*f*-electrons) and nearly-free conduction bands (*c*-electrons). The interplay between the *f* and *c*-electrons is theorized to give rise to local moment formation^4,20–25^, and to cascades between light and heavy fermions at integer moiré fillings^3^. However, direct evidence for local moment formation and associated heavy fermion behavior, remains lacking.

In this work, we employ planar tunneling spectroscopy (PTS)^26,27^ to measure the inverse compressibility, d*μ*/d*n*, from which we extract the chemical potential, μ, electronic entropy, S, and effective mass, ${m}^{ * }$. Our measurements reveal two entropy plateaus at 10 K and 20 K, whose combinatorial degeneracy marks the emergence of 4-fold and 8-fold isospin degenerate local moment states, respectively. The 4-fold degenerate state arises from strain-induced degeneracy lifting of the eight heavy fermion flavors. We find distinct ground states across different moiré band fillings and uncover mass renormalization transitions between heavy and light fermions that persist up to $\sim 70\,{{{\rm{K}}}}$. These transitions are well described by a Kondo lattice model featuring localized *f*-electrons at AA sites coexisting with itinerant *c-*electrons^24,25^. Near moiré filling of $n/{n}_{0}=1$, we find that the inverse compressibility shows a pronounced non-monotonic temperature dependence, reflecting a transition from a low-temperature gapless heavy Fermi liquid to a high-temperature, high-entropy, solid-like local moment state. Here n is the carrier density and ${n}_{0}$ corresponds to one electron per moiré cell. The non-monotonic temperature dependence of the inverse compressibility may stem from the emergence of the Kondo effect at low temperatures or from the presence of a Dirac node associated with a potential symmetry-breaking state, i.e., the incommensurate Kekulé spiral state.

## Results

### Planar tunneling spectroscopy and electronic compressibility

Planar tunneling spectroscopy (PTS) provides access to key electronic properties - including energy gaps, mid-gap states, and quasiparticle excitations - with a mechanically rigid and highly reproducible tunneling junction. Compared to conventional scanning tunneling spectroscopy (STS), PTS offers several advantages: significantly enhanced junction stability, superior energy resolution, and straightforward compatibility with ultra-low temperatures and high magnetic fields in standard dilution-refrigerator environments. Crucially, PTS also enables gate-voltage spectroscopy, $\frac{{{{\rm{d}}}}I}{{{{\rm{d}}}}{V}_{g}}$, with I the tunneling current and ${V}_{g}$ the gate voltage, which is inaccessible in STS because the feedback-controlled junction cannot maintain the required stability under gate modulation.

**Fig. 1:**
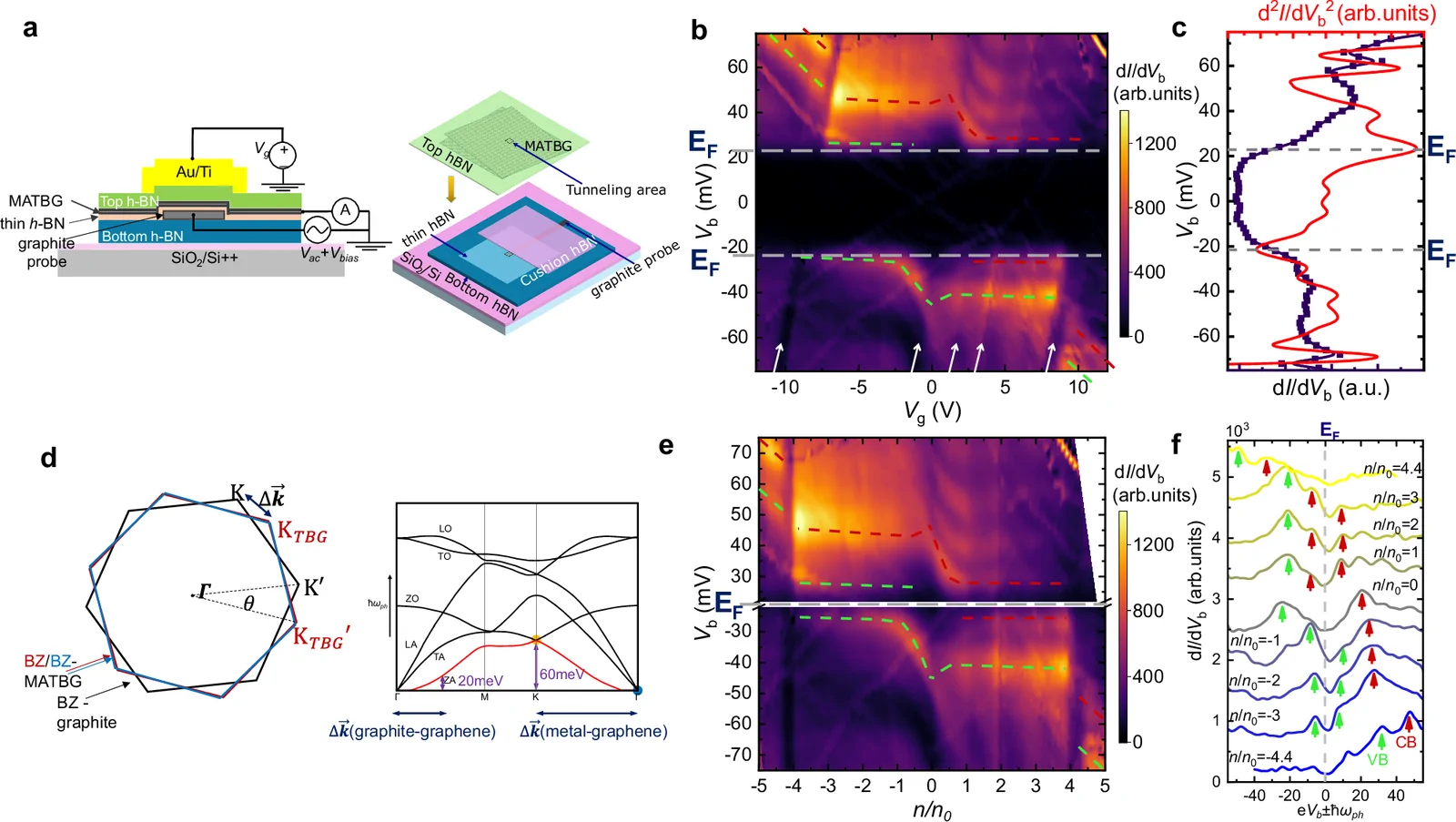
Device structure and d*I*/d*V*_*b*_ map. **a** Schematic diagram of the device structure (Left) and stacking order of the van der Waals heterostructures (Right), showing the planar tunneling measurement configuration with a graphite probe. The AC voltage can be applied to either the bias voltage (as shown in the diagram) or the gate voltage. See Methods for details. **b** ${{{\rm{d}}}}I/{{{\rm{d}}}}{V}_{b}$ of 1.08° MATBG (${V}_{{ac}}=0.8 \, {{{\rm{mV}}}}$, 11.07 Hz) as a function of ${V}_{b}$ and ${V}_{g}$. Red and green dashed lines mark the conduction and valence flat bands. Dark tilted lines indicating the suppressed dI/dV_b_ are marked by orange arrows. **c** ${{{\rm{d}}}}I/{{{\rm{d}}}}{V}_{b}$ and ${{{{\rm{d}}}}}^{2}I/{{{\rm{d}}}}{V}_{b}^{2}$ at ${V}_{g}=-10 \, {{{\rm{V}}}}$. Gray dashed lines mark the onset of inelastic tunneling channels and the Fermi level at ${V}_{{{{\rm{b}}}}}=\pm 22 \, {{{\rm{mV}}}}$. This onset voltage is independent of ${V}_{g}$. **d** Left: Reciprocal-space geometry of MATBG and graphite probe. The momentum mismatch between the sample and the probe due to an 18° orientation difference can be bridged at a higher energy by absorbing or emitting a phonon. Right: Phonon spectrum of graphite. The blue solid dot represents electrons in a metal probe. The orange solid dot represents electrons in TBG. The momentum and energy required from the phonon are labeled for tunneling between the metal probe and TBG, and for tunneling between the graphite probe and TBG on the right and left, respectively. **e** Same as **b**, but sheared to show filling dependence and normalized by the transmission coefficient as discussed in the text. The phonon-induced gap feature near ${V}_{{{{\rm{b}}}}}=0 \, {{{\rm{mV}}}}$ is omitted. Red and green dashed lines mark the conduction and valence flat bands. Suppressed ${{{\rm{d}}}}I/{{{\rm{d}}}}{V}_{b}$ is observed at $n/{n}_{0}=\pm 4$. **f** ${{{\rm{d}}}}I/{{{\rm{d}}}}{V}_{b}$ spectra at indicated fillings. The peaks indicating conduction band (CB), and valance band (VB) are marked by red and green arrows.

The planar tunneling device used here (Fig. 1a) consists of a thin (2 nm) hexagonal boron nitride (h-BN) flake serving as the tunneling barrier, a narrow (1 μm) graphite ribbon serving as the tunneling probe, and a top gate separated from the MATBG sample by a thicker (30 nm) h-BN layer (Methods). The fixed probe-sample distance in PTS ensures a constant tunneling gap, eliminating variations caused by mechanical vibrations or electrostatic forces during gate and temperature sweeps. This stability enables high energy resolution and reliable measurement of the electronic inverse compressibility and chemical potential as a function of temperature and magnetic field (Methods). The twist angle in the tunneling area of our device, 1.06° ± 0.02°, is determined by calibrating the carrier density, n, using high magnetic field Landau levels (LLs) and Chern insulators (CIs) (Methods).

Figure 1b shows the bias and gate voltage dependence (${V}_{b}$ and ${V}_{g}$, respectively) of the differential conductance $\frac{{{{\rm{d}}}}I}{{{{\rm{d}}}}{V}_{b}}$ measured at 0.3 K. Near ${V}_{b}=0 \, {{\rm{mV}}}$, we observe a gap-like suppression of $\frac{{{{\rm{d}}}}I}{{{{\rm{d}}}}{V}_{b}}$ that persist across all ${V}_{g}$ values. This feature, commonly referred to as the “phonon gap”, is well known from STS measurements of graphene. It arises from the momentum mismatch between electrons tunneling from a metallic tip, which originate near the Γ -point (${{{\boldsymbol{k}}}}\approx 0$), and graphene’s low-energy states located at the ${{{\rm{ K}}}}$ point, whose momentum magnitude is $\left|{{{\boldsymbol{K}}}}\right|=\frac{4\pi }{3a}=1.7{{{{{\AA }}}}}^{-1}$, with *a* = 0.246 nm the graphene lattice constant. Because direct elastic tunneling is suppressed, electrons can tunnel into a graphene layer either via an inelastic process involving a ~60 mV flexural phonon that supplies the needed momentum transfer^28–30^, or by using an atomically sharp STM tip that naturally emits electrons with higher transverse momentum^31,32^. Here we use graphite as the tunneling probe, with its lattice orientation misaligned with that of the TBG sample by $\sim 18^\circ$ as determined from AFM imaging (SI Appendix B). This misalignment produces a momentum mismatch $\Delta {{{\boldsymbol{k}}}}=\left|{{{{\boldsymbol{K}}}}}_{{{{\boldsymbol{g}}}}}-{{{{\boldsymbol{K}}}}}_{{TBG}}\right|\approx 0.53 \, {{{{{\AA }}}}}^{-1}$ which suppresses elastic tunneling and requires an inelastic process involving a ~20 meV acoustic (ZA-branch) phonon to provide the necessary momentum transfer (Fig. 1d). This gives rise to the characteristic phonon gap, visible as the dark central strip in the $\frac{{{{\rm{d}}}}I}{{{{\rm{d}}}}{V}_{b}}$ map (Fig. 1b). From the $\frac{{{{\rm{d}}}}I}{{{{\rm{d}}}}{V}_{b}}$ spectrum and its derivative, $\frac{{{{{\rm{d}}}}}^{2}I}{{{{\rm{d}}}}{V}_{b}^{2}}$, at ${V}_{g}=-10 \, {{{\rm{V}}}}$ (Fig. 1c) we find that the gap is symmetric about ${V}_{b}=0 \, {{\rm{mV}}}$ and is flanked by sharp peaks in $\frac{{{{{\rm{d}}}}}^{2}I}{{{{\rm{d}}}}{V}_{b}^{2}}$ at $\pm 22 \, {{{\rm{mV}}}}$, corresponding to the onset of the phonon-assisted tunneling channels^33^. The $\pm 22 \, {{{\rm{mV}}}}$ threshold is consistent with the phonon energy required to overcome the momentum mismatch of $\sim 0.53 \, {{{{{\AA }}}}}^{-1}$ (Fig. 1d).

Because elastic tunneling is suppressed within the gap, the effective Fermi level, ${E}_{F}$, is pinned at the onset of inelastic tunneling on both electron and hole sides. Thus ${E}_{F}=\mu \left(n\right) \sim \pm 22 \, {{{\rm{mV}}}}$, flanks the gap symmetrically as indicated by the dashed white lines in Fig. 1b. Here $\mu \left(n\right)$, the chemical potential measured relative to the charge-neutrality point, is identified with ${E}_{F}$.

The red and green dashed lines in Fig. 1b trace the peaks in the differential conductance, which follow the gate-dependent energies associated with the maxima in the density of states (DOS) of the conduction and valence bands, respectively. When the active MATBG bands are either empty (${V}_{g} < -8 \, {{{\rm{V}}}}$) or full (${V}_{g} > +8 \, {{{\rm{V}}}}$), these two features remain parallel to each other, separated by $\sim 16 \, {{{\rm{mV}}}}$, while shifting strongly with gate voltage. In the partially filled regime ($-8 \, {{{\rm{V}}}} < {V}_{g} < +8 \, {{{\rm{V}}}}$) and away from charge neutrality (${V}_{g} \sim 0 \, {{\rm{V}}}$) their energies become nearly independent of gate voltage (horizontal dashed lines) and exhibit a $\sim 15 \, {{{\rm{mV}}}}$ splitting at the Fermi level (Fig. 1e). Near the CNP the dashed lines develop a finite slope, signaling the opening of a gap consistent with previous STS measurements^14^.

Figure 1b also shows a set of dark tilted lines (marked by white arrows) along which $\frac{{{{\rm{d}}}}I}{{{{\rm{d}}}}{V}_{b}}$ is suppressed up to high bias voltages. Their slope, which reflects how the carrier density depends simultaneously on ${V}_{g}$ and ${V}_{b}$, is determined by the capacitance ratio $\frac{{C}_{g}}{{C}_{b}} \sim \frac{1}{27}$, where ${C}_{g}$ and ${C}_{b}$ denote the capacitances between the sample and the gate and between the sample and the bias electrode, respectively. For this geometry, the total induced charge in the sample is ${ne}={C}_{g}{V}_{g}-{C}_{b}{V}_{b}$, where e is the electron charge. This differs from the common approximation used in transport and STM/STS experiments, ${ne}\approx {C}_{g}{V}_{g}$, where the contribution from ${C}_{b}$ is negligible due to the large probe sample distance in transport and small probe area in STS.

The “sheared” $\frac{{{{\rm{d}}}}I}{{{{\rm{d}}}}{V}_{b}}$ map in Fig. 1e presents the same $\frac{{{{\rm{d}}}}I}{{{{\rm{d}}}}{V}_{{{{\rm{b}}}}}}$ data as in Fig. 1b, but plotted as a function of the carrier density per moiré cell, $n/{n}_{0}$, rather than gate voltage ${V}_{g}$. Expressing the data in terms of $n/{n}_{0}$ removes the explicit ${V}_{b}$ dependence of *n*, causing the previously tilted features to appear as vertical dark lines at the integer moiré fillings $n/{n}_{0}=\pm 4$ and 0^34^. In addition, as discussed below, the data was normalized to remove the non-intrinsic density dependence introduced by the transmission coefficient, $T\left(n\right)$. Figure 1f shows the individual spectra at several fillings, with the conduction and valence band peaks marked by red and green arrows, respectively.

To analyze the data, we model the tunneling current using the standard assumptions of a constant probe DOS (Methods) and an energy-independent, but filling-dependent, transmission coefficient $T\left(n\right)$^34^. The tunneling current is then,

1 $$I={I}_{0}T\left(n\right){\int }_{\mu \left(n\right)}^{\mu \left(n\right)+{{eV}}_{b}}\rho \left(\epsilon \right){{{\rm{d}}}}\epsilon,$$

where $\rho \left(\epsilon \right)$ is the sample DOS, $\mu \left(n\right)$ is the chemical potential, and ${I}_{0}$ is a constant prefactor.

To isolate the contribution from $\mu \left(n\right)$ we divide the measured current by the independently characterized transmission coefficient, *T*(*n*)$\propto \exp \left(0.258\cdot n/{n}_{0}\right)$, which accounts for the dependence of the tunneling barrier on the carrier density (SI Appendix C). Defining the normalized current ${I}_{n}=\frac{I}{T\left(n\right)}$ and using ${E}_{F}=\mu \left(n\right)$, we differentiate with respect to ${V}_{b}$ to obtain the bias dependent differential conductance (Methods):

2 $${\left.\frac{{{{\rm{d}}}}{I}_{n}}{{{{\rm{d}}}}{V}_{b}}\right|}_{{V}_{b}}=e{I}_{0}\left[\rho \left({E}_{F}+e{V}_{b}\right)-\frac{d\mu \left(n\right)}{{dn}}\frac{{C}_{b}}{{e}^{2}}\left(\rho \left({E}_{F}+e{V}_{b}\right)-\rho \left({E}_{F}\right)\right)\right].$$

Here ${C}_{b}=e\partial n/\partial {V}_{b}$ is the tip-sample capacitance. The first term, $\rho \left({E}_{F}+e{V}_{b}\right)$, is the usual DOS term familiar from conventional STS. The second term, which contains $\frac{{{{\rm{d}}}}\mu }{{{{\rm{d}}}}n}$, is normally negligible in STS because the tip–sample capacitance is extremely small. In contrast, in our planar tunneling geometry, the tip-sample capacitance is large (${C}_{b} \sim 1.3{{{\rm{\mu }}}}{{{\rm{F}}}}/{{{{\rm{cm}}}}}^{2}$), making this term observable. However, since both $\frac{{{{\rm{d}}}}\mu }{{{{\rm{d}}}}n}$ and ρ contribute to the measured $\frac{{{{\rm{d}}}}{I}_{n}}{{{{\rm{d}}}}{V}_{b}}$, an additional measurement is required to isolate $\frac{{{{\rm{d}}}}\mu }{{{{\rm{d}}}}n}$. This independent handle comes from the gate-dependent differential conductance $\frac{{{{\rm{d}}}}I}{{{{\rm{d}}}}{V}_{g}}$. The exceptional stability of the planar tunneling junction enables simultaneous measurement of $\frac{{{{\rm{d}}}}I}{{{{\rm{d}}}}{V}_{g}}$, which also depends on both ρ and $\frac{{{{\rm{d}}}}\mu }{{{{\rm{d}}}}n}$, allowing us to extract $\frac{{{{\rm{d}}}}\mu }{{{{\rm{d}}}}n}$ reliably. Differentiating Eq. (1) with respect to ${V}_{g}$ while keeping ${V}_{b}$ fixed yields

3 $$\frac{{{{\rm{d}}}}{I}_{n}}{{{{\rm{d}}}}{V}_{g}}={eI}_{0}\frac{{{{\rm{d}}}}\mu \left(n\right)}{{dn}}\frac{{C}_{g}}{{e}^{2}}\left(\rho \left(\mu \left(n\right)+e{V}_{b}\right)-\rho (\mu (n))\right).$$

where ${C}_{g}=e\partial n/\partial {V}_{g}$ is the gate capacitance.

Equations (2) and (3) together provide two independent observables that depend differently on the DOS and on $\frac{{{{\rm{d}}}}\mu }{{{{\rm{d}}}}n}$. This enables a direct and robust extraction of $\frac{{{{\rm{d}}}}\mu }{{{{\rm{d}}}}n}$ from the combined bias and gate dependent tunneling measurements (Methods):

4 $${\left.\frac{{{{\rm{d}}}}\mu \left(n\right)}{{{{\rm{d}}}}n}\right|}_{{V}_{b}}=\frac{{e}^{2}\frac{{{{\rm{d}}}}{I}_{n}}{{{{\rm{d}}}}{V}_{g}}}{{C}_{g}\frac{{{{\rm{d}}}}{I}_{n}}{{{{\rm{d}}}}{V}_{b}}+{C}_{b}\frac{{{{\rm{d}}}}{I}_{n}}{{{{\rm{d}}}}{V}_{g}}}.$$

**Fig. 2:**
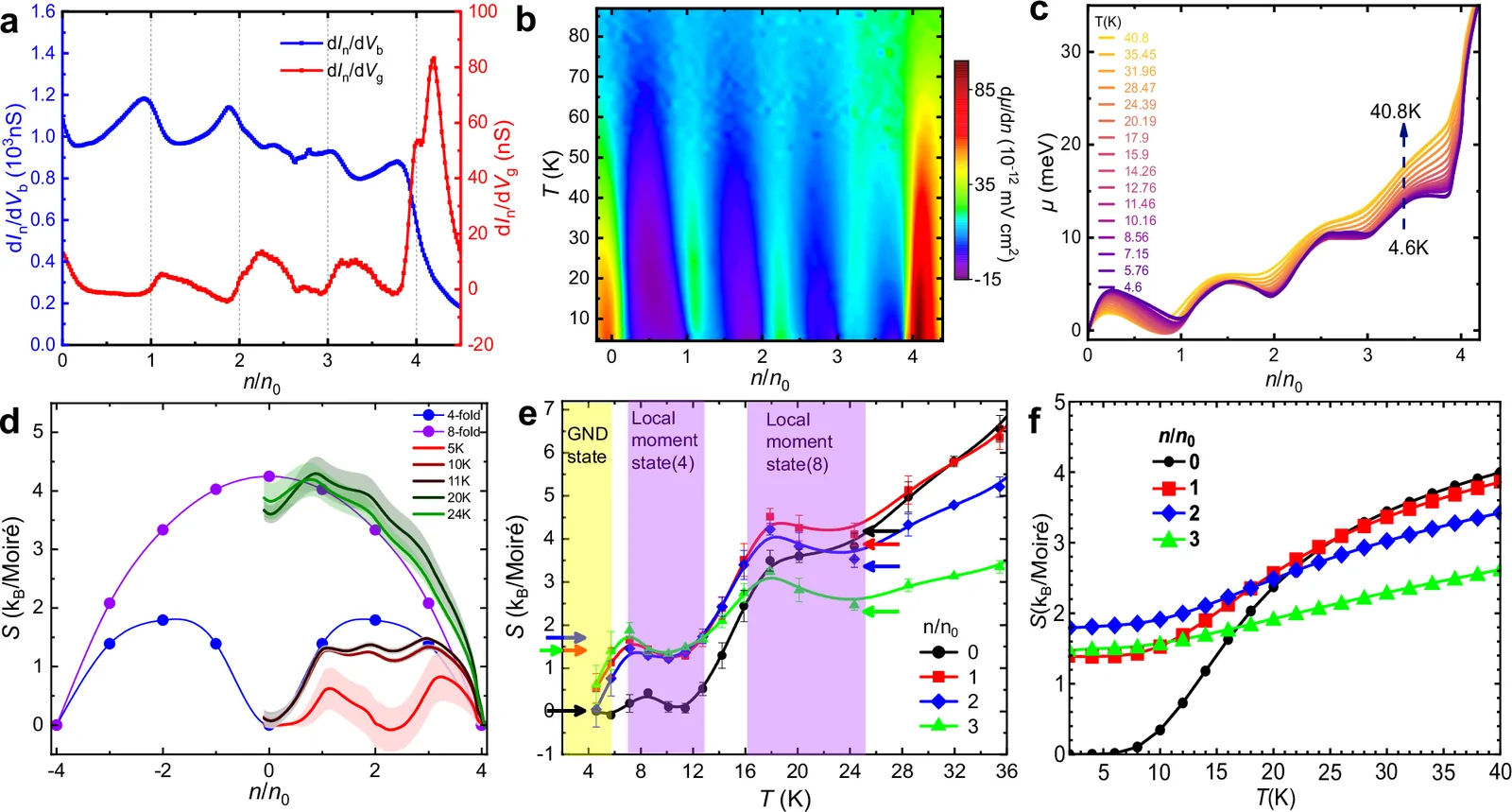
Temperature dependence of inverse compressibility and entropy. **a** ${{{\rm{d}}}}{I}_{n}/{{{\rm{d}}}}{V}_{b}$ and ${{{\rm{d}}}}{I}_{n}/{{{\rm{d}}}}{V}_{g}$ on the electron side taken at ${V}_{b}=-42 \, {{{\rm{mV}}}}$ at 4 K. **b** ${{{\rm{d}}}}\mu /{{{\rm{d}}}}n$ map on the electron side as a function of filling and temperature showing a cascade of transitions near integer fillings that persist up to 70 K. **c** Temperature and filling dependence of chemical potential μ calculated by integrating ${{{\rm{d}}}}\mu /{{{\rm{d}}}}n$. **d** Filling dependence of electronic entropy at various temperatures. The entropy (solid lines) is obtained by solving Maxwell’s relation for every three adjacent temperature data points. The shaded area around the curve represents the error bars, indicating the estimated 1-sigma (1σ) uncertainty of the fitted entropy. Blue and purple dots are calculated by counting all possible microstates with degeneracy of 4 and 8, respectively. **e** Temperature dependence of entropy at non-zero integer fillings Two plateaus are observed near 10 K and 20 K. Left facing and right facing arrows indicate the theoretical 8 and 4 microstate entropy respectively, for fillings 0,1,2,3 marked by black, red, blue, and green symbols, respectively. Error bars represent the estimated 1σ uncertainty of the fitted entropy. **f** Entropy at integer fillings calculated in the atomic limit of the topological heavy-fermion model with strain. The Hubbard interaction energy U_1_ of 23 mV and the compressive heterostrain ε=0.001 are set to match the experimental data (see SI Appendix I). Below and above 15 K, the entropy shows two plateaus corresponding to the degeneracy of 4 and 8, in good agreement with the measured entropy. Note that in the atomic limit, the *f*-electrons at different lattice sites are decoupled from one another and from the *c*-electrons, which is not valid for the real ground states at low temperatures below 6 K.

Using this procedure, we obtained the temperature and filling dependent inverse compressibility at high temperatures, $10 \, {{{\rm{K}}}}-70 \, {{{\rm{K}}}}$, and down to the base temperature $0.3 \, {{{\rm{K}}}}$ (Fig. 2b and Fig. S2). We then integrated the inverse compressibility over the carrier density to obtain the chemical potential relative to the CNP and its temperature dependence,

$\mu \left(n/{n}_{0},T\right)={\int }_{0}^{\frac{n}{{n}_{0}}}\left(\frac{{{{\rm{d}}}}\mu \left(n,T\right)}{{{{\rm{d}}}}n}\right){{{\rm{d}}}}n,$ shown in Fig. 2c. To obtain the correct carrier density and filling values we included the quantum capacitance contribution (Methods, SI Appendix D).

### Entropy spectroscopy and combinatorial degeneracy of local moment states

Figure 2b shows the evolution of $\frac{{{{\rm{d}}}}\mu }{{{{\rm{d}}}}n}\left(\frac{n}{{n}_{0}},T\right)$ on the electron side, over the temperature range $4 \, {{{\rm{K}}}}-85 \, {{{\rm{K}}}}$. Using Maxwell’s relation $\frac{\partial S}{\partial n}=-\frac{\partial \mu }{\partial T}$, and assuming that the entropy per moiré cell is zero at full filling (due to the band gap between the flat and remote band) we extract the entropy $S\left(\frac{n}{{n}_{0}},T\right)=-{\int }_{4}^{\frac{n}{{n}_{0}}}{\left(\frac{\partial \mu }{\partial T}\right)}_{\frac{n}{{n}_{0}}}{{{\rm{d}}}}\left(\frac{n}{{n}_{0}}\right)$, from the measured temperature dependent chemical potential, shown in Fig. 2c (Methods).

In Fig. 2d we compare the experimentally extracted entropy $S\left(\frac{n}{{n}_{0}}\right)$ with the expected filling-dependent entropy calculated by counting all possible microstates assuming degeneracies of 4 (blue dots) and 8 (purple dots). Below $20 \, {{{\rm{K}}}}$ the data show a clear transition from the high temperature 8-fold degeneracy to a 4-fold degeneracy, signaling the emergence of a broken symmetry state. To further investigate this transition, Fig. 2e presents the temperature dependence of the entropy at moiré fillings $n/n_{0}={{\mathrm{0,1,2}}}$, and 3, over the temperature range $4.2 \, {{{\rm{K}}}}$ to $36 \, {{{\rm{K}}}}$. Consistent with previous work^35,36^, the entropy increases with temperature. But in addition, owing to the high resolution of our PTS technique, we are able to resolve two distinct entropy plateaus: one near $10 \, {{{\rm{K}}}}$ and another near $20 \, {{{\rm{K}}}}$ (Fig. 2e). These plateaus align well with the formation of two solid-like local moment states: a high temperature state of *f*-electrons carrying randomly oriented 8-fold degenerate isospin moments and low temperature broken symmetry state where the isospin degeneracy is reduced from 8 to 4.^21^.

Within the framework of Kondo lattice models^20–25^, at relatively high temperatures the conduction (*c*) and localized (*f*) fermions behave as decoupled subsystems. In this so-called zero-hybridization limit (SI Appendix I)^24^, electrons occupy the *f*-fermion states at integer fillings while the *c*-electron sector remains half-filled. This results in a localized, solid-like lattice of *f*-electrons at the AA moiré sites, each hosting random isospin local moments that combine spin, valley, and sublattice degrees of freedom.

The presence of two plateaus, rather than one, suggests that the degeneracy of these local moments changes between $10 \, {{{\rm{K}}}}$ and $20 \, {{{\rm{K}}}}$. Comparison of the filling-dependent entropy data with a simple combinatorial model of integer-filled *f*-electrons carrying random local moments (Fig. 2d and SI Appendix H) shows quantitative agreement. Around $20 \, {{{\rm{K}}}}$, corresponding to the high-temperature plateau, the entropy values closely match those expected for an eightfold degeneracy, $$S={k}_{B}{{\mathrm{ln}}}\left(\begin{array}{c}8\\ n/{n}_{0}+4\end{array}\right)$$, as shown more clearly in Fig. 2e by the left facing arrows (black, red, blue, green arrows corresponding to fillings 0,1,2,3 respectively). Near $10 \, {{{\rm{K}}}}$— the low-temperature plateau—the reduced entropy corresponds to a fourfold degeneracy  $$S={k}_{B}{{\mathrm{ln}}}\left(\begin{array}{c}4\\ |n/{n}_{0}|\end{array}\right)$$, shown by the color matched right facing arrows in Fig. 2e.

This interpretation is further supported by the entropy behavior at the CNP which saturates to zero below $12 \, {{{\rm{K}}}}$ (Fig. 2d, e), consistent with the absence of local moments there. The observed change in degeneracy is consistent with broken symmetries, possibly strain induced^37^, which is known to be present in these systems. Figure 2d shows the total entropy of *f*- and *c*-electrons at integer fillings, calculated using the zero-hybridization limit of the topological heavy-fermion (THF) model, including strain effects (SI Appendix I)^4,37^. This model captures the key experimental features—the two entropy plateaus and their magnitudes—supporting the interpretation of isospin local moments with combinatorial degeneracies. Since the simplified model ignores *f-c* hybridization and correlations between AA sites, the calculated entropy does not approach zero at low temperatures as seen experimentally, and as expected for heavy Fermi liquid or for correlated symmetry-broken insulators. Upon heating, the calculated entropy saturates at the second plateau, but only at temperatures above $20{{{\rm{K}}}}$. The absence of further increase at higher temperatures is likely due to the omission of additional phonon contributions or *f-c* hybridization effects (SI Appendix J)^21,37^.

Theoretically, the entropy at $n/{n}_{0}=2$ should be higher than that at $n/{n}_{0}=1$ or 3 due to the larger combinatorial degeneracy. Experimentally, however, we observe that the entropy at $n/{n}_{0}=2$ is significantly reduced below $\sim 5 \, {{{\rm{K}}}}$ compared to the other fillings, with the difference diminishing as temperature rises to $\sim 8 \, {{{\rm{K}}}}$ (Fig. 2d, e). This temperature-dependent deviation from non-interacting theory reflects the emergence of correlated insulating behavior at $n/{n}_{0}=2$, as discussed later.

### Compressibility and mass renormalization signatures of *f*–*c* electron interplay

**Fig. 3:**
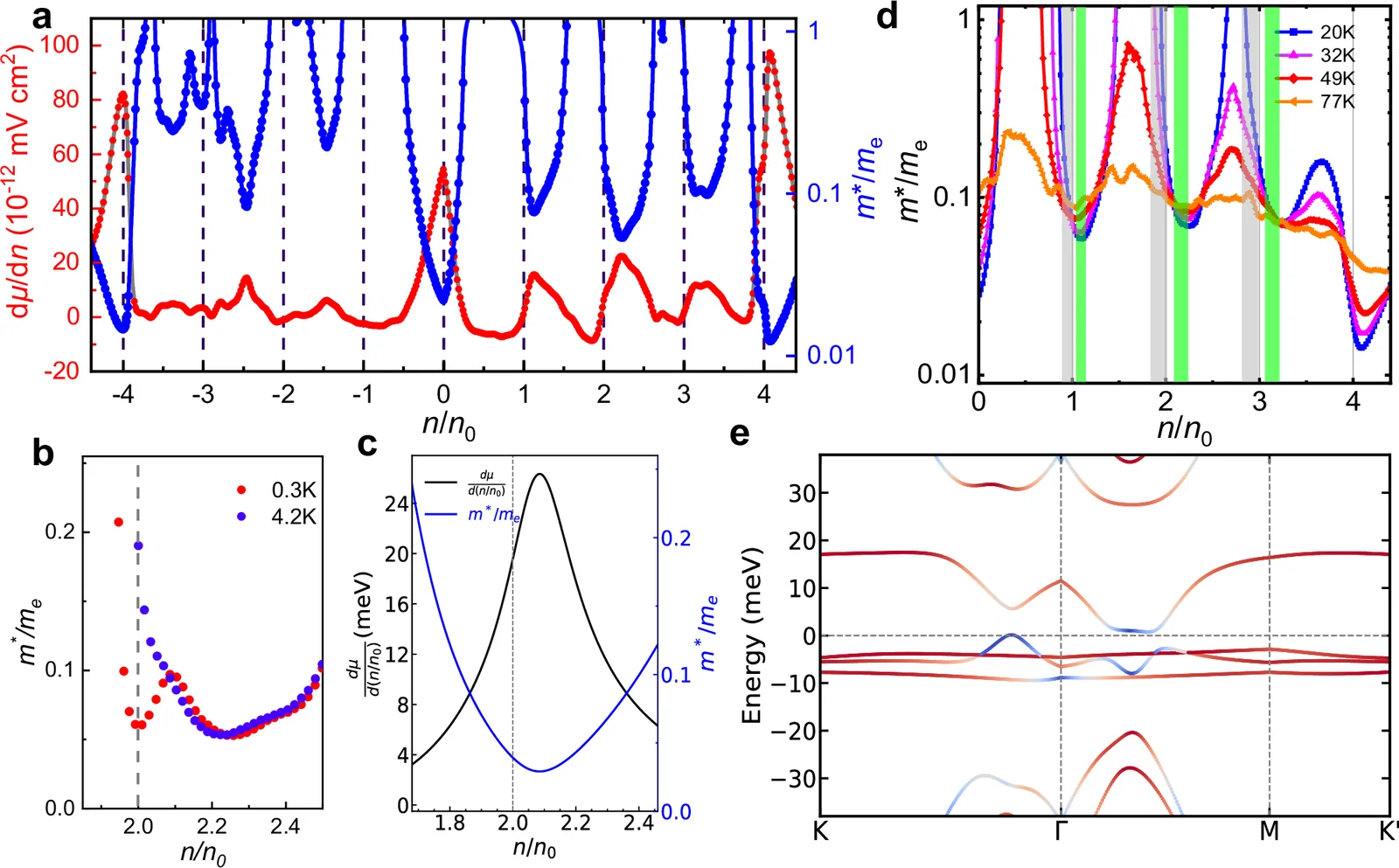
Heavy fermion and mass renormalizations at integer fillings. **a** ${{{\rm{d}}}}\mu /{{{\rm{d}}}}n$ and cyclotron effective mass ${m}^{*}$ in units of electron mass *m*_*e*_ as a function of $n/{n}_{0}$ at 4 K. Upon approaching each positive integer filling 1, 2, and 3, ${m}^{*}$ drops from high to low, reaching a minimum at filling of $n/{n}_{0}+\delta$. Shaded rectangles mark regions where ${{{\rm{d}}}}\mu /{{{\rm{d}}}}n$ turns negative and m^*^ cannot be extracted using Eq. 4. **b** Cyclotron effective mass ${m}^{*}/{m}_{e}$ near $n/{n}_{0}=2$ at 0.3 K and 4 K. **c** Theoretical calculation of ${{{\rm{d}}}}\mu /{{{\rm{d}}}}(n/{n}_{0})$ and ${m}^{*}/{m}_{e}$ near $n/{n}_{0}=2$ at $T=20 \, {{{\rm{K}}}}$ for compressive heterostrain ε=0.001 (see SI Appendix G) **d** Cyclotron effective mass ${m}^{*}/{m}_{e}$ above 20 K. Prominent cascades of transitions from heavy (gray shade) to light (green shade) across integer fillings survive until $\sim 77 \, {{{\rm{K}}}}$. **e** Calculated band structure of topological heavy-fermion model for IKS state at filling 2 (ε=0.001, see SI Appendix G): *f*-orbitals (red) and *c*-orbitals (blue). With hybridization, charge-one excitation bands are formed having both *f*- and *c*- character.

The low temperature electronic inverse compressibility, $\frac{{{{\rm{d}}}}\mu }{{{{\rm{d}}}}n}$, shown in Fig. 3a exhibits pronounced filling-dependent variations. It changes from low or even negative values on the lower-doping side of positive integer fillings to sharp peaks on the higher-doping side. Similar non-monotonic behavior of the flat bands has been observed with other techniques and attributed to a sequence of Fermi surface reconstructions^14,17–19^. As $\frac{n}{{n}_{0}}$ approaches each non-zero integer filling from below, $\frac{{{{\rm{d}}}}\mu }{{{{\rm{d}}}}n}$ turns negative, indicating the emergence of a more stable lower energy ground state. Upon crossing an integer filling, $\frac{{{{\rm{d}}}}\mu }{{{{\rm{d}}}}n}$ rises sharply to a peak before decreasing and turning negative again as the filling approaches the next integer.

This pattern – where a peak in $\frac{{{{\rm{d}}}}\mu }{{{{\rm{d}}}}n}$ follows a negative dip - resembles the correlation gap characteristic of Mott insulators^38^. Here, however, it is attributed to the transition from heavy to light quasiparticles across the integer filling.

Using the measured $\frac{{{{\rm{d}}}}\mu }{{{{\rm{d}}}}n}$, we extract the effective cyclotron mass ${m}^{ * }$ and its filling dependence. The cyclotron effective mass for a differentiable region of the band structure is defined as ${m}^{ * }=\frac{{\hslash }^{2}}{2\pi }\frac{\partial A}{\partial E}$, where ℏ is the reduced Planck constant, and *A* is the Fermi surface area. For a two-dimensional system without strong correlations, ${m}^{ * }$ relates to the inverse compressibility by

5 $${m}^{ * }=\frac{2\pi {\hslash }^{2}}{g^{\prime} }{\left(\frac{{{{\rm{d}}}}\mu }{{{{\rm{d}}}}n}\right)}^{-1},$$

where $g^{\prime}=4$ accounts for spin and valley degeneracy.

Figure 3a shows both $\frac{{{{\rm{d}}}}\mu }{{{{\rm{d}}}}n}$ and normalized effective mass ${m}^{ * }/{{m}_{e}}$ (${m}_{e}$ the bare electron mass) at $4 \, {{{\rm{K}}}}$ across the full flat band filling range. Remarkably, near positive integer fillings ($n/{n}_{0}={{\mathrm{1,2,3}}}$), ${m}^{ * }/{{m}_{e}}$ varies substantially with filling, from values exceeding 1 just below integer filings to as low as ~0.06 just above integer filling where it approaches values comparable to bilayer graphene (0.03-0.05)^39^. This asymmetry is consistent with recent Seeback experiments reporting heavy (light) excitations below (above) positive integer fillings^40^.

The effective mass calculation, as defined by Eq. (5), is only valid as long as $\frac{{{{\rm{d}}}}\mu }{{{{\rm{d}}}}n}$ is positive. A negative inverse compressibility reflects strong interaction effects such as exchange-correlation contributions, partial subsystem occupancy, or incipient phase separation^41^ and is not directly relatable to m^*^. Therefore, at low temperatures in regions where $\frac{{{{\rm{d}}}}\mu }{{{{\rm{d}}}}n}$ is negative (gray rectangles in Fig. 3a), we cannot extract the value of m^*^. Near the CNP, where this method fails due to the gap opening, we used the Landau Level fans to obtain ${m}^{ * }/{{m}_{e}}=0.174$ (Methods).

**Fig. 4:**
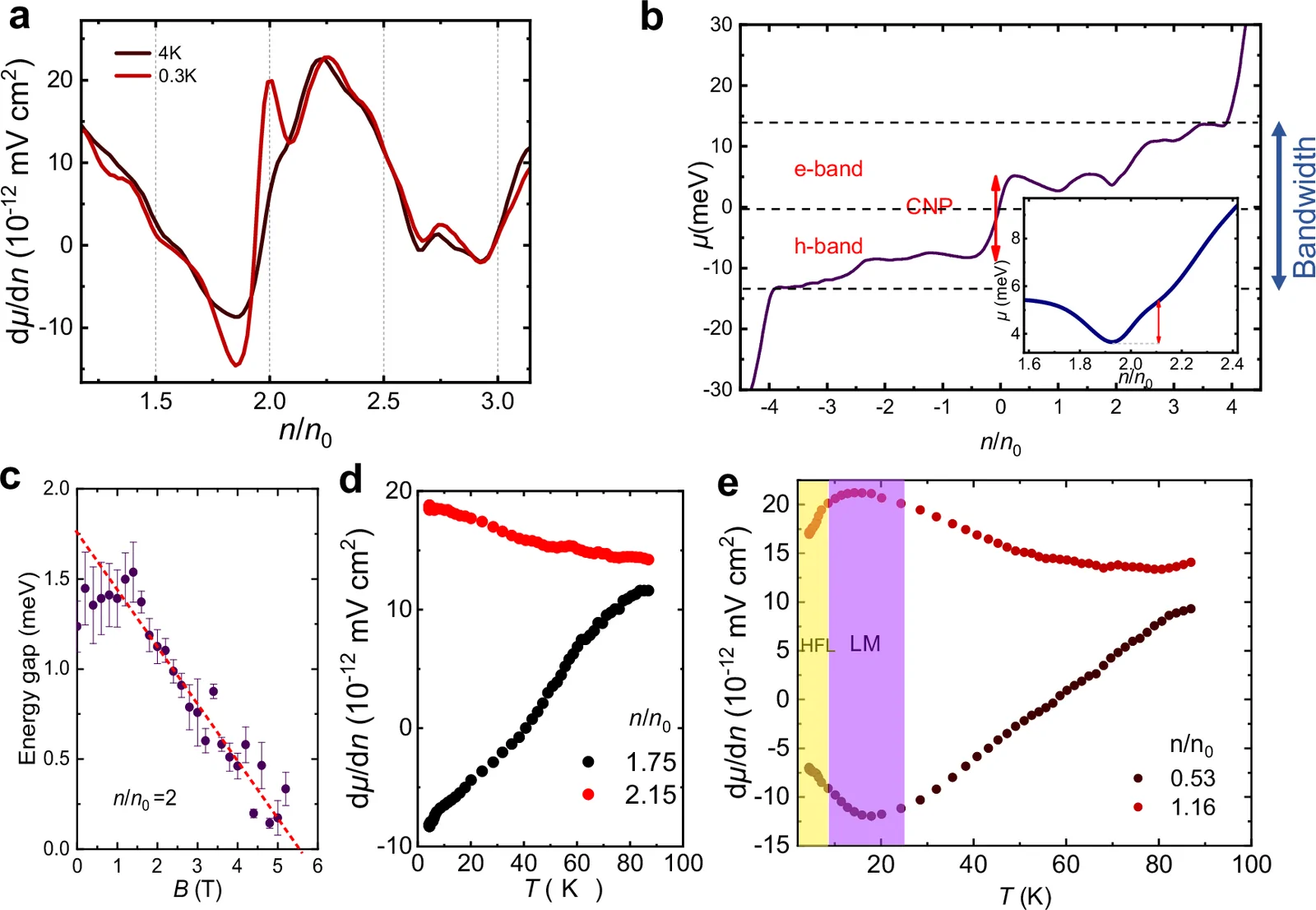
Electronic inverse compressibility and magnetic field dependence. **a** ${{{\rm{d}}}}\mu /{{{\rm{d}}}}n$ at $0.3 \, {{{\rm{K}}}}$ and $4 \, {{{\rm{K}}}}$ near filling of 2 showing the opening of a gap right at $n/{n}_{0}=2$ below 4 K. **b** Chemical potential μ as function of filling integrated from $d\mu /{dn}$ at 0.3K. Chemical potential jump (red arrow) indicates a $10 \, {{{\rm{meV}}}}$ gap at the CNP. Inset, zoom-in around filling 2 shows a $\sim 1.3 \, {{{\rm{meV}}}}$ gap at $n/{n}_{0}=2$ (red arrow). **c** Gap (jump in chemical potential) at $n/{n}_{0}=2$ versus perpendicular magnetic field. The red dashed line is a linear fit for $B > 1.6 \, {{{\rm{T}}}}$ yielding a g-factor of 5.4. The error bars represent the estimated variations of the energy gaps. **d** Monotonic temperature dependence of $d\mu /{dn}$ near $n/{n}_{0}=2$. **e** Non-monotonic temperature dependence of ${{{\rm{d}}}}\mu /{{{\rm{d}}}}n$ near $n/{n}_{0}=1$.

In Fig. 3b we show the filling dependence of ${m}^{ * }/{{m}_{e}}$ near $n/{n}_{0}=2$. for $T=4.2 \, {{{\rm{K}}}}$ and $0.3 \, {{{\rm{K}}}}$, At $T=4.2 \, {{{\rm{K}}}}$, ${m}^{ * }/{{m}_{e}}$ decreases monotonically from a value of 0.2 at $n/{n}_{0}=2$, to a minimum of 0.06 at $n/{n}_{0}=2.1$, whereas at $T=0.3 \, {{{\rm{K}}}}$ where a gap opens (Fig. 4a), the behavior is nonmonotonic displaying a sharp dip at $n/{n}_{0}=2$ and then recovering to join the curve at 4.2 K.

Hartree-Fock simulations in the symmetry-broken phase (Fig. 3c) mirror the experimental trends of the 4.2 K data.

We note that the strong mass renormalization of both electron and hole excitations is evident near all integer fillings and persists up to temperatures as high as $70 \, {{{\rm{K}}}}$ (Fig. 3d). The pronounced filling dependence of ${m}^{ * }$ is consistent with theoretical expectations for both symmetry-broken^3,4,42,43^ and symmetric^25,44,45^ phases of MATBG. Such behavior arises naturally within the THF model^4,21,23–25,44,46,47^ and related approaches^5,6,20,45^ in which the active moiré bands emerge from the hybridization between localized, nearly dispersionless *f*-electrons - experimentally observed via STM/STS to occupy the AA moiré lattice^7,18^- and itinerant *c*-electrons. Within this framework, hole-like excitations near integer fillings acquire predominantly *f*-electron character, while electron-like excitations remain largely *c*-like. This asymmetry in orbital composition naturally produces the strongly asymmetric behavior observed in both $\frac{{{{\rm{d}}}}\mu }{{{{\rm{d}}}}n}$ and ${m}^{ * }$. As illustrated by the THF band structure calculation at filling 2 (Fig. 3e) for a symmetry-broken incommensurate Kekulé spiral (IKS) ground state (SI Appendix G), the *f*-electron character (blue) dominates the hole sector just below the integer filling, whereas doping above integer filling populates electron-like states with predominantly *c*-electron character (red). This agreement between theory and experiment provides strong evidence that the mass renormalization reflects the interplay between *f*-electrons and itinerant *c*-electrons.

### Correlation induced gap and Pomeranchuk effect

By cooling from $4 \, {{{\rm{K}}}}$ down to our base temperature, $0.3 \, {{{\rm{K}}}}$, we find that the inverse compressibility remains essentially unchanged for most fillings (SI Appendix A). A notable exception occurs at $n/{n}_{0}=2$ where at $0.3 \, {{{\rm{K}}}}$ a sharp peak in $\frac{{{{\rm{d}}}}\mu }{{{{\rm{d}}}}n}$ accompanied by a dip below the same filling, (Fig. 4a) reveals the emergence of a correlation induced gap. This behavior is fully consistent with the reduced entropy (Fig. 2d) and the reduced effective mass (Fig. 3b) observed at this filling. The filling dependent chemical potential, $\mu \left(n/{n}_{0}\right),$ obtained by integrating the $\frac{{{{\rm{d}}}}\mu }{{{{\rm{d}}}}n}$ curves at 0.3 K (Fig. 4b) reveals a flat-band bandwidth of $\sim 26 \, {{{\rm{meV}}}}$ and a $10 \, {{{\rm{meV}}}}$ gap at the CNP, extracted from the discontinuity in μ (red arrow). Zooming in to $n/{n}_{0}=2$, (Fig. 4b inset), we observe another clear jump in the chemical potential, which we attribute to a combination of correlation-induced gap formation and band reconstruction. From the change in μ across the filling range $1.95\le n/{{n}_{0}}\le 2.1$, we estimate a gap $\Delta=1.3\pm 0.14 \, {{{\rm{meV}}}}$, in good agreement with transport measurements^17^ and scanning single-electron transistor studies^48^. To understand the nature of the correlated state at $n/{n}_{0}=2$, we examine the magnetic field dependence of $\frac{{{{\rm{d}}}}\mu }{{{{\rm{d}}}}n}$ in the electron sector at $0.3 \, {{{\rm{K}}}}$ (SI Appendix A, hole sector discussed in the SI Appendix F). The field dependence of the gap at $n/{n}_{0}=2$ (Fig. 4c) shows that above $1.5 \, {{{\rm{T}}}}$, the gap decreases linearly with field, indicating a spin-unpolarized ground state. This is consistent with the intervalley coherent spin singlet phase previously inferred from transport, where strong resetting of the Hall number occurs when six of the eight bands (two bands per flavor) are filled and the remaining two are empty^17^. A linear fit of $\Delta={\Delta }_{0}-g{\mu }_{{{{\rm{B}}}}}B$ yields a g-factor of 5.4±0.2, pointing to a substantial orbital contribution to the magnetic moment. Here ${\Delta }_{0}=1.75 \, {{{\rm{meV}}}}$ and ${\mu }_{{{{\rm{B}}}}}$ is the Bohr magneton. Below $1.5 \, {{{\rm{T}}}}$, the gap remains essentially constant. The experimentally observed properties of the emergent low temperature correlated state at $n/{n}_{0}=2$ are fully consistent with theoretical predictions of the incommensurate Kekulé spiral (IKS) state^37,49^, a symmetry-broken phase proposed to arise in the presence of strain and also observed in STM experiments^50^. In sharp contrast to filling 2, where the gapped symmetry-broken state is the ground state, the situation at filling 1 is markedly different. The $\frac{{{{\rm{d}}}}\mu }{{{{\rm{d}}}}n}$ signal near filling 1 (Fig. 4e) exhibits a pronounced non-monotonic temperature dependence, with a maximum (at $n/{n}_{0}=0.54$) and a minimum (at $n/{n}_{0}=1.16$) both appearing near $16 \, {{{\rm{K}}}}$. This behavior provides direct thermodynamic evidence for a Pomeranchuk-like effect, complementing earlier indications based on resistance measurements^35,36^. By contrast, the inverse compressibility near filling 2 remains monotonic with temperature (Fig. 4d), underscoring the fundamentally different physics of the two states.

The data suggest that at low temperatures, the $n/{n}_{0}=1$ phase is a gapless liquid state. One possible explanation for the observed temperature dependence of $\frac{{{{\rm{d}}}}\mu }{{{{\rm{d}}}}n}$ invokes Kondo physics: upon cooling below $\sim 16 \, {{{\rm{K}}}}$, the system crosses over from a high-temperature local-moment regime to a low-temperature heavy Fermi-liquid state, where additional *f*-electron spectral weight emerges near the Fermi energy, reducing $\frac{{{{\rm{d}}}}\mu }{{{{\rm{d}}}}n}$. Alternatively, the behavior may reflect a gapless symmetry-broken IKS state at low temperatures, characterized by a Dirac node lying close to the Fermi energy. Both scenarios are compatible with a gapless, highly renormalized state, consistent with the thermodynamic signatures observed near filling 1.

## Discussion

This work establishes a quantitative experimental basis for heavy fermion behavior coupled with nontrivial topology in twisted bilayer graphene. Through the observed combinatorial degeneracy of isospin local moments and its characteristic 4/8 transition, we identify an entropic contribution in Kondo systems that points toward a strain-mediated pathway for symmetry breaking in topological correlated materials

Furthermore, we identify clear signatures of strong mass enhancement consistent with hybridization between localized and itinerant electrons in a Kondo-lattice framework. Finally, the pronounced non-monotonic temperature dependence of $\frac{{{{\rm{d}}}}\mu }{{{{\rm{d}}}}n}$ near filling 1 indicates a crossover from a low-temperature heavy Fermi liquid region to a high-temperature local-moment regime, consistent with a Pomeranchuk-like entropy-driven effect.

## Methods

### Sample fabrication

The planar tunneling stacks were prepared using dry transfer and ‘tear and stack’ methods^51^ in a dry argon atmosphere inside a glovebox. A narrow graphite ribbon (~1 μm wide, ~3 nm thick) was first exfoliated onto a PMMA film and then dry-transferred onto a bottom h-BN flake ($30-70 \, {{{\rm{nm}}}}$ thick) exfoliated on a SiO_2_/Si substrate. An ultra-thin h-BN sheet (2-6 layers thick) serving as the tunneling layer was then exfoliated and dry-transferred to cover one end of the graphite probe. The remaining area was covered with another cushion h-BN flake (5−10 nm thick) to prevent unwanted tunneling, forming a probing area of $\sim 1 \, {\mathrm{\mu m}}\times 1 \, {\mathrm{\mu m}}$. The entire bottom stack was annealed at 300 °C in forming gas (10% H_2_ and 90% Ar) overnight to remove PMMA residue. The top stack was constructed by first picking up a top h-BN flake ($30-50 \, {{{\rm{nm}}}}$ thick) using a PDMS/PPC hemispherical handle. A large monolayer graphene, exfoliated on SiO_2_/Si substrate, was then picked up in halves by the h-BN flake with a twist angle of 1.1° between two layers. The h-BN/TBG top stack was finally released onto the bottom stack at around 90 °C, aligning the bubble-free TBG region with the small tunneling probe. An Au top gate was deposited over the TBG area for electrical gating. Electrical edge contacts (Cr/Au) were deposited after e-beam lithography and etching with CHF_3_ plasma^52^. The temperature during fabrication was kept below 160 °C to prevent possible relaxation of TBG.

### Planar tunneling measurements

The planar tunneling measurements were performed in a ^3^He refrigerator at a base temperature of 0.3 K, with a maximum perpendicular magnetic field of 8 T. We have used a calibrated on-chip thermometer (Cernox CX−1010 calibrated 0.3–292 K) and an on-chip heater to ensure accurate, local temperature readout and control throughout the measurements. The TBG was electrically gated using a gate voltage ${V}_{g}$ applied to the Au top gate relative to the sample, and the tunneling current was driven by a bias voltage ${V}_{b}$ applied to the sample relative to the tunneling probe. Differential conductance curves were measured using the standard lock-in technique, with an AC bias voltage modulation ($0.2-1 \, {{{\rm{mV}}}}$, $11.07 \, {{{\rm{Hz}}}}$) superimposed on the DC ${V}_{b}$, or with an AC gate voltage modulation ($5-10 \, {{{\rm{mV}}}}$, $11.07 \, {{{\rm{Hz}}}}$) superimposed on the DC ${V}_{g}$. The tunneling current was amplified by an external current preamplifier with a gain of ${10}^{9}\frac{{{{\rm{V}}}}}{{{{\rm{A}}}}}$ and measured at the same frequency as the AC voltage modulation. Unlike the reported inverse compressibility and entropy measurements^35,36^, our approach directly resolves the DOS with higher energy resolution (370 μeV at 0.3 K) and enables measurements over a broader and more finely resolved temperature range.

### Quantum capacitance correction to carrier density calculation

An example of extracting $\frac{{{{\rm{d}}}}\mu }{{{{\rm{d}}}}n}$ on the electron side at ${V}_{b}=-42 \, {{\rm{mV}}}$ and 4 K curves is shown in Fig. S1b. We note that the $\frac{{{{\rm{d}}}}\mu }{{{{\rm{d}}}}n}$ peaks corresponding to the expected band gaps at fillings ±4 are shifted away by about 0.2. This discrepancy arises when the carrier density is calculated based on the geometric capacitance ${C}_{{geo}}\approx {C}_{g}$ alone while neglecting the contribution of the quantum capacitance. In fact, the geometric capacitance and quantum capacitance, *C*_*q*_, are connected in series, so that the actual carrier density at a given ${V}_{g}$ and ${V}_{b}=0$ is ${ne}={\left(\frac{1}{{C}_{g}}+\frac{1}{{C}_{q}}\right)}^{-1}{V}_{{{{\rm{g}}}}}$. While ${C}_{g}$ is a known constant determined by sample geometry, ${C}_{q}$ is proportional to the DOS. Therefore, when the sample enters insulating states at band edges, the quantum capacitance contribution is not negligible, and the filling calculation must be adjusted. All the $\frac{{{{\rm{d}}}}\mu }{{{{\rm{d}}}}n}$ results shown in figures were first calculated assuming a constant total capacitance ${\left(\frac{1}{{C}_{{geo}}}+\frac{1}{{C}_{q}}\right)}^{-1}$. After applying the correction (SI Appendix C), the $\frac{d\mu }{{dn}}$ peaks marking the band edges separating the flat bands from remote bands, appear at $n/{n}_{0}=\pm 4$, as shown in Fig. 2b. Away from the band edges, within the flat band, the quantum capacitance contribution is negligible, as illustrated in Fig. S8a (SI Appendix D).

### Carrier density per moiré cell and twist angle

To accurately determine the carrier density and twist angle we use high magnetic field Landau levels (LLs) and Chern insulators (CIs) (Fig.S2b, S2c, SI Appendix A). Using the LL fan at the CNP, $n\left({V}_{{{{\rm{g}}}}}\right)={\nu }_{{LL}}(B/{\phi }_{0}),{{{\rm{where}}}} \, {\nu }_{{LL}}={{\mathrm{4,8,12}}}\ldots$ is the LL filling factor and ${\phi }_{0}$ the flux quantum, we obtain *n* = 0.30 × 10^12^ ${V}_{g}$ (cm^−2^). From this relation, together with the trajectories of Chern insulators which follow the Streda formula $n\left({V}_{g}\right)=s{n}_{0}+{\nu }_{{LL}}\left(B/{\phi }_{0}\right)$ where $s,{\nu }_{{LL}}{\mathbb{\in }}{\mathbb{Z}}$ and, we obtain *n*_0_ = 0.67 × 10^12^ cm^−2^ and the twist angle in radians $\theta=a{\left(\frac{2}{\sqrt{3}{n}_{0}}\right)}^{\tfrac{1}{2}}$, which corresponds to 1.07°.

### Normalized tunneling current and differential conductance

We analyze the measured tunneling current, $I={I}_{0}{\int }_{{E}_{F}}^{{E}_{F}+{{eV}}_{b}}T\left(n\right)\rho \left(\epsilon \right){{{\rm{d}}}}\epsilon$, under the assumption that the probe DOS and transmission coefficient, $T\left(n\right)$, are energy-independent^27,31,34,37^. Here, we comment on the assumption of a constant DOS in the graphite probe. Due to the interlayer coupling in graphite, there is an overlap of approximately 0.7 eV between the valence and conduction bands near the Fermi level, resulting in the absence of a bandgap and giving graphite its semi-metallic character^53,54^. As a result, the DOS is low compared to metal, but much higher than that of few-layer graphene. Since STM/STS is highly surface-sensitive and primarily probes the electronic structure of the top few layers, it does not directly reflect the bulk DOS of graphite. In our experiment, the graphite probe is approximately 3 nm thick and thus already exhibits bulk-like electronic properties. Although the DOS varies with energy, it can be reasonably approximated as constant within a narrow energy window around the Fermi level^55^. In our case, the relevant energy range is less than 70 mV. Similar treatments could also be found in the planar tunneling spectroscopy of other reports^56^. We further simplify the expression by dividing it by an exponential transmission coefficient background $T\left(n\right) \sim \exp \left(-\frac{2d}{\hslash }\sqrt{2m\Delta \left({{n}}\right)}\right)$^57,58^ (SI Appendix B) resulting from the barrier height dependence on carrier density, $\Delta\left({{n}}\right)$. This gives the normalized tunneling current ${I}_{n}=\frac{I}{T\left(n\right)}={I}_{0}{\int }_{{E}_{F}}^{{E}_{F}+{{eV}}_{b}}\rho \left(\epsilon \right){{{\rm{d}}}}\epsilon,$ where ${I}_{0}$ is a constant. Taking the derivative with respect to ${V}_{b}$, and recalling that ${E}_{F}=\mu \left(n\right)$, we obtain

6 $$\frac{{{{\rm{d}}}}{I}_{n}}{{{{\rm{d}}}}{V}_{b}}={I}_{0}\left[\left(\frac{\partial \left(\mu \left(n\right)+e{V}_{b}\right)}{\partial {V}_{b}}\right)\rho \left(\mu \left(n\right)+e{V}_{b}\right)-\frac{\partial \mu \left(n\right)}{\partial {V}_{b}}\rho (\mu (n))\right]$$

$$=e{I}_{0}\left[\rho \left({E}_{F}+e{V}_{b}\right)-\frac{{{{\rm{d}}}}\mu \left(n\right)}{{{{\rm{d}}}}n}\frac{{C}_{b}}{{e}^{2}}\left(\rho \left({E}_{F}+e{V}_{b}\right)-\rho \left({E}_{F}\right)\right)\right]$$

Where ${C}_{b}=e\partial n/\partial {V}_{b} \sim 1.3{{{\rm{uF}}}}/{{{{{\rm{cm}}}}}^{2}}$ is the capacitance between the sample and the bias electrode.

In our planar tunneling structure, the ultra-stable tunneling junction enables us to measure the derivative of the tunneling current with respect to the gate voltage $({V}_{g})$

$$\frac{{{{\rm{d}}}}{I}_{n}}{{{{\rm{d}}}}{V}_{g}}={I}_{0}\left[\frac{\partial \mu \left(n\right)}{\partial {V}_{g}} \rho \left(\mu \left(n\right)+e{V}_{b}\right)-\frac{\partial \mu \left(n\right)}{\partial {V}_{g}} \rho \left(\mu \left(n\right)\right)\right]$$

which simplifies to:

7 $$\frac{{{{\rm{d}}}}{I}_{n}}{{{{\rm{d}}}}{V}_{g}}={eI}_{0}\frac{{{{\rm{d}}}}\mu \left(n\right)}{{{{\rm{d}}}}n}\frac{{C}_{g}}{{e}^{2}}\left(\rho \left(\mu \left(n\right)+e{V}_{b}\right)-\rho \left(\mu \left(n\right)\right)\right).$$

By solving Eqs. (6) and (7), we arrive at the following expressions:

8 $$\rho \left(\mu \left(n\right)+e{V}_{b}\right)=\tfrac{\tfrac{{{{\rm{d}}}}{I}_{n}}{{{{\rm{d}}}}{V}_{b}}+\tfrac{{C}_{b}}{{C}_{g}}\tfrac{{{{\rm{d}}}}{I}_{n}}{{{{\rm{d}}}}{V}_{g}}}{e{I}_{0}},$$

9 $$\frac{{{{\rm{d}}}}\mu \left(n\right)}{{{{\rm{d}}}}n}=\frac{e\frac{d{I}_{n}}{d{V}_{g}}\frac{1}{{{I}_{0}C}_{g}}}{\rho \left(\mu \left(n\right)+e{V}_{b}\right)-\rho \left(\mu \left(n\right)\right)},$$

where ${C}_{g}$ is the capacitance between the sample and the gate electrode.

### Determination of effective mass at CNP and remote bands through Landau levels

Due to suppressed kinetic energy and a small bandwidth of the flat bands, Landau levels (LLs) in these bands are closely spaced and difficult to observe in energy spectra. However, from our planar tunneling results --- specifically, the high $\frac{{{{\rm{d}}}}\mu }{{{{\rm{d}}}}n}$ and the appearance of dark lines under perpendicular magnetic fields --- we identify elevated $\frac{d\mu }{{dn}}$ at doping levels corresponding to gaps between LLs, with a Landau filling factor ${\nu }_{{LL}}=0,\pm 4,\pm 8,\pm 12$, and a degeneracy of 4 near the CNP. This observation is consistent with transport measurements that show ${R}_{{{{\rm{xx}}}}}$ dips and quantized ${R}_{{{{\rm{xy}}}}}$. In contrast, the remote bands, which have a larger Fermi velocity, are expected to display more pronounced LLs. This is evident in the $\frac{{{{\rm{d}}}}I}{{{{\rm{d}}}}{V}_{b}}$ map under a magnetic field of 6 T (SI Appendix E).

To study the LLs of the flat bands in detail, we conducted high-resolution $\frac{{{{\rm{d}}}}I}{{{{\rm{d}}}}{V}_{b}\left({V}_{b},\frac{n}{{n}_{0}}\right)}$ map measurements at a base temperature of 0.3 K near the CNP. The Landau filling factor ${\nu }_{{LL}}$ can be calculated using the Streda formula: $n/{n}_{0}\left(s,{\nu }_{{LL}}\right)=s+{\nu }_{{LL}}(\phi /{\phi }_{0})\,{{{\rm{where}}}} \, s,{\nu }_{{LL}}{\mathbb{\in }}{\mathbb{Z}}$. In Figure S3a, S3b (SI Appendix A) where the $\frac{{{{\rm{d}}}}I}{{{{\rm{d}}}}{V}_{b}}$ is plotted at a fixed ${V}_{b}$ from 0 T to 2 T, LLs with ${\nu }_{{LL}}=\pm 12,\pm 8,\pm 4,0$ emerge at fields as low as 0.6 T. Above 1.5 T, all the degeneracies of the 0^th^ LL are lifted, consistent with the previous transport measurements^17^. The pattern reveals a Landau fan similar to that seen in transport measurements^17^, but here the gaps between LLs or Chern bands correspond to $\frac{d\mu }{{dn}}$ maxima rather than to the ${R}_{{{{\rm{xx}}}}}$ minima. The direct correspondence between the vertical dark lines in the $\frac{{{{\rm{d}}}}I}{{{{\rm{d}}}}{V}_{b}}$ map and the $\frac{{{{\rm{d}}}}\mu }{{{{\rm{d}}}}n}$ peaks is illustrated through a side-by-side comparison of the $\frac{{{{\rm{d}}}}I}{{{{\rm{d}}}}{V}_{b}\left({V}_{b},\frac{n}{{n}_{0}}\right)}$ and $\frac{{{{\rm{d}}}}\mu }{{{{\rm{d}}}}n}\left(\frac{n}{{n}_{0}}\right)$ without and with magnetic field (SI Appendix E). Near the CNP, we observe LLs (Fig. S3, SI Appendix A) whose 4-fold degeneracy is lifted above 3.5 T, revealing a full LL fan emanating from the 0^th^ LL and half fans emerging from fillings 1, 2, and 3. The latter replicate those identified in transport measurements as Chern insulators states^17^. We calculate their Chern numbers using the Streda formula: $C={\nu }_{{LL}}=\frac{n/{n}_{0}-s}{(\phi /{\phi }_{0})}$ ${{{\rm{where}}}}$ ${{{\rm{s}}}}=\pm 1,\pm 2,\pm 3$ is the band index and $s\cdot {\nu }_{{LL}} > 0$^15,17,59,60^. In Fig.4e, the filling-dependent chemical potential curves illustrate the evolution of LLs and CIs with the magnetic field. The differential conductance with background subtraction $\frac{{{{\rm{d}}}}I}{{{{\rm{d}}}}{V}_{b}^{ * }}$, is plotted as a function of ${V}_{b}-{V}_{D}$ (${V}_{D}$ is the bias voltage at the Dirac point) from 1 T to 3 T, highlighting LLs on the electron side (Fig. S3c, SI Appendix A). The background is defined as an average of the $\frac{{{{\rm{d}}}}I}{{{{\rm{d}}}}{V}_{b}}$ spectra over the Landau filling ${\nu }_{{LL}}$. The energy difference between LLs follows a linear relation with the magnetic field, characteristic of massive Dirac fermions, rather than the square root dependence typical of massless Dirac fermions. Both the Landau filling sequence and this linear dependence support the presence of massive chiral bands near the CNP. By fitting the LLs of the flat bands to the energy expression for massive Dirac fermion ${E}_{N}-{E}_{0}=\hslash {\omega }_{c}\sqrt{N\left(N-1\right)}$ we extract an effective mass ${m}^{ * }=0.174 \, {m}_{e}$.

A similar analysis of the LLs for remote bands is shown in Fig. S3d (SI Appendix A). Near the CNP, LLs for those bands appear at higher energy, starting at 60 mV above 1 T. The bias voltage of the LL peaks for both flat and remote bands as a function of $\sqrt{N\left(N+1\right)}B$ is shown in Fig. S3e. From the energy expression for the N^th^ LL relative to the Dirac point ${E}_{N}-{E}_{0}=\hslash {\omega }_{c}\sqrt{N\left(N-1\right)}$, where ${\omega }_{c}=\frac{{eB}}{{m}^{ * }}$, the slope is inversely proportional to the effective mass. The remote bands have a much lighter mass, ${m}^{ * }=0.047 \, {m}_{e}$, than the flat bands, consistent with the expectation of dispersive remote bands and flat central bands. However, note that this estimation of ${m}^{ * }$ for flat bands applies to low fillings near the CNP. As the system crosses integer fillings, it undergoes strong transitions from heavy to light effective mass.

### Extraction of entropy from T-dependent chemical potential

The entropy is calculated from temperature-dependent chemical potential results (Figure S4a, SI Appendix A) by integrating the Maxwell’s relation

10 $${\left(\frac{\partial S}{\partial \nu }\right)}_{T}=-{\left(\frac{\partial \mu }{\partial T}\right)}_{\nu },$$

where S is the entropy per moiré unit cell, μ is the chemical potential, T is the temperature, and $\nu \equiv \frac{n}{{n}_{0}}$ is the moiré filling factor. Similar to previous single-electron transistor experiments^35,36^, we fix the chemical potential at the CNP to 0 and assume that the entropy at filling of 4 is 0 due to the large band gap separating the flat bands from the remote bands.

11 $$S\left(\frac{n}{{n}_{0}},T\right)=-\int _{4}^{\frac{n}{{n}_{0}}}{\left(\frac{\partial \mu }{\partial T}\right)}_{\frac{n}{{n}_{0}}}{{{\rm{d}}}}\left(\frac{n}{{n}_{0}}\right).$$

To minimize the error, we switch the order of integration and differentiation, which does not affect the entropy calculation:

12 $$S\left(\frac{n}{{n}_{0}},T\right)=-\frac{\partial {\int }_{4}^{\frac{n}{{n}_{0}}}{\mu }_{\frac{n}{{n}_{0}}}{{{\rm{d}}}}\left(\frac{n}{{n}_{0}}\right)}{\partial T}.$$

The integral of the chemical potential $-{\int }_{4}^{\frac{n}{{n}_{0}}}{\mu }_{\frac{n}{{n}_{0}}}{{{\rm{d}}}}\left(\frac{n}{{n}_{0}}\right)$ for fillings of 2 and 3, from 4 K to 40 K is shown in Fig. S5b (SI Appendix A). The temperature-dependent upward drift of the chemical potential at filling of 4 is consistent with the results reported in both SET experiments^35^ and graphene sensing experiments^16^. This is consistent with the broadening of the central flat bands as the temperature is increased until eventually they are no longer isolated from remote bands. The entropy values and corresponding errors are obtained by performing a linear fit using three adjacent temperature points, as displayed in Fig. S5c (SI Appendix A).

## Supplementary information

Supplementary Information

Peer Review file

## Source data

Source Data 1

Source Data 2

Source Data 3

Source Data 4

## Data Availability

The data that support the findings of this work are available from the corresponding author upon request. Source data is provided with the paper. Source data are provided with this paper.

## References

[1] Coleman, P. Heavy Fermions: Electrons at the Edge of Magnetism. *in Handbook of Magnetism and Advanced Magnetic Materials*, (2007).

[2] Stewart, G. R. Heavy-fermion systems. *Rev. Mod. Phys.* **56**, 755–787 (1984).

[3] Kang, J., Bernevig, B. A. & Vafek, O. Cascades between Light and Heavy Fermions in the Normal State of Magic-Angle Twisted Bilayer Graphene. *Phys. Rev. Lett.* **127**, 266402 (2021). 10.1103/PhysRevLett.127.266402 35029496

[4] Song, Z. D. & B. A. Bernevig, magic-angle twisted bilayer graphene as a topological heavy fermion problem. *Phys. Rev. Lett.* **129** 10.1103/PhysRevLett.129.047601 (2022). 35939005

[5] Shi, H. & Dai, X. Heavy-fermion representation for twisted bilayer graphene systems. *Phys. Rev. B* **106**, 245129 (2022).

[6] Haule, M. & Haule, E. Y. A., K. The *Mott-semiconducting state in the magic angle bilayer graphene*. arXiv:1901.09853, (2019).

[7] Li, G. et al. Observation of Van Hove singularities in twisted graphene layers. *Nat. Phys.* **6**, 109–113 (2010).

[8] Bistritzer, R. & MacDonald, A. H. Moire bands in twisted double-layer graphene. *Proc. Natl. Acad. Sci. USA* **108**, 12233–12237 (2011). 10.1073/pnas.1108174108 PMC3145708 21730173

[9] Andrei, E. Y. & MacDonald, A. H. Graphene bilayers with a twist. *Nat. Mater.* **19**, 1265–1275 (2020). 10.1038/s41563-020-00840-0 33208935

[10] Cao, Y. et al. Unconventional superconductivity in magic-angle graphene superlattices. *Nature* **556**, 43 (2018). 10.1038/nature26160 29512651

[11] Cao, Y. et al. Nematicity and competing orders in superconducting magic-angle graphene. *Science* **372**, 264–271 (2021). 10.1126/science.abc2836 33859029

[12] Cao, Y. et al. Correlated insulator behaviour at half-filling in magic-angle graphene superlattices. *Nature* **556**, 80 (2018). 10.1038/nature26154 29512654

[13] Cao, Y. et al. Strange Metal in Magic-Angle Graphene with near Planckian Dissipation. *Phys. Rev. Lett.* **124**, 076801 (2020). 10.1103/PhysRevLett.124.076801 32142336

[14] Jiang, Y. H. et al. Charge order and broken rotational symmetry in magic-angle twisted bilayer graphene. *Nature* **573**, 91 (2019). 10.1038/s41586-019-1460-4 31365921

[15] Nuckolls, K. P. et al. Strongly correlated Chern insulators in magic-angle twisted bilayer graphene. *Nature* **588**, 610–615 (2020). 10.1038/s41586-020-3028-8 33318688

[16] Park, J. M. et al. Flavour Hund’s coupling, Chern gaps and charge diffusivity in moiré graphene. *Nature* **592**, 43–48 (2021). 10.1038/s41586-021-03366-w 33790447

[17] Wu, S. et al. Chern insulators, van Hove singularities and topological flat bands in magic-angle twisted bilayer graphene. *Nat. Mater.* **20**, 488–494 (2021). 10.1038/s41563-020-00911-2 33589799

[18] Wong, D. L. et al. Cascade of electronic transitions in magic-angle twisted bilayer graphene. *Nature* **582**, 198 (2020). 10.1038/s41586-020-2339-0 32528095

[19] Zondiner, U. et al. Cascade of phase transitions and Dirac revivals in magic-angle graphene. *Nature* **582**, 203 (2020). 10.1038/s41586-020-2373-y 32528091

[20] Datta, A. et al. Heavy quasiparticles and cascades without symmetry breaking in twisted bilayer graphene. *Nat. Commun.* **14**, 5036 (2023). 10.1038/s41467-023-40754-4 PMC10439139 37596252

[21] Zhou, G. D. et al. Kondo phase in twisted bilayer graphene. *Phys. Rev. B.* **109**, 10.1103/PhysRevB.109.045419 (2024).

[22] Lau, L. L. H. & Coleman, P. Topological Mixed Valence Model for Twisted Bilayer Graphene. *Phys. Rev. X*. **15**, 021028 (2025).

[23] Hu, H. Y. et al. Symmetric Kondo Lattice States in Doped Strained Twisted Bilayer Graphene. *Phys. Rev. Lett.* **131**, 10.1103/PhysRevLett.131.166501 (2023). 10.1103/PhysRevLett.131.166501 37925696

[24] Hu, H. Y., Bernevig, B. A. & Tsvelik, A. M. Kondo Lattice Model of Magic-Angle Twisted-Bilayer Graphene: Hund’s Rule, Local-Moment Fluctuations, and Low-Energy Effective Theory. *Phys. Rev. Lett.* **131**, 10.1103/PhysRevLett.131.026502 (2023). 37505959

[25] Chou, Y. Z. & Das Sarma, S. Kondo Lattice Model in Magic-Angle Twisted Bilayer Graphene. *Phys. Rev. Lett.* **131**, 10.1103/PhysRevLett.131.026501 (2023). 37505969

[26] Britnell, L. et al. Electron tunneling through ultrathin boron nitride crystalline barriers. *Nano Lett.* **12**, 1707–1710 (2012). 10.1021/nl3002205 22380756

[27] Dvir, T. et al. Spectroscopy of bulk and few-layer superconducting NbSe_2_ with van der Waals tunnel junctions. *Nat. Commun.* **9**, 598 (2018). 10.1038/s41467-018-03000-w PMC5807409 29426840

[28] Zhang, Y. B. et al. Giant phonon-induced conductance in scanning tunnelling spectroscopy of gate-tunable graphene. *Nat. Phys.* **4**, 627–630 (2008).

[29] Chandni, U. et al. Signatures of phonon and defect-assisted tunneling in planar metal-hexagonal boron nitride-graphene junctions. *Nano Lett.* **16**, 7982–7987 (2016). 10.1021/acs.nanolett.6b04369 27960492

[30] Inbar, A. et al. The quantum twisting microscope. *Nature* **614**, 682–687 (2023). 10.1038/s41586-022-05685-y 36813895

[31] Li, G., Luican, A. & Andrei, E. Y. Scanning tunneling spectroscopy of graphene on graphite. *Phys. Rev. Lett.* **102**, 176804 (2009). 10.1103/PhysRevLett.102.176804 19518809

[32] Lu, C.-P. et al. Local, global, and nonlinear screening in twisted double-layer graphene. *Proc. Natl. Acad. Sci.* **113**, 6623–6628 (2016). 10.1073/pnas.1606278113 PMC4914180 27302949

[33] Mohr, M. et al. Phonon dispersion of graphite by inelastic x-ray scattering. *Physical Review B.* **76** 10.1103/PhysRevB.76.035439 (2007).

[34] Malec, C. E. & Davidovic, D. Transport in graphene tunnel junctions. *J. Appl. Phys.* **109** 10.1063/1.3554480 (2011).

[35] Saito, Y. et al. Isospin Pomeranchuk effect in twisted bilayer graphene. *Nature* **592**, 220 (2021). 10.1038/s41586-021-03409-2 33828322

[36] Rozen, A. et al. Entropic evidence for a Pomeranchuk effect in magic-angle graphene. *Nature* **592**, 214-+ (2021). 10.1038/s41586-021-03319-3 33828314

[37] Herzog-Arbeitman, J. et al. Kekul\‘e spiral order from strained topological heavy fermions. *Phys. Rev. B* **112**, 125129 (2025).

[38] Kotliar, G., Murthy, S. & Rozenberg, M. J. Compressibility divergence and the finite temperature mott transition. *Phys. Rev. Lett.* **89**, 046401 (2002). 10.1103/PhysRevLett.89.046401 12144491

[39] Zou, K., Hong X. & Zhu, J. Effective mass of electrons and holes in bilayer graphene: Electron-hole asymmetry and electron-electron interaction. *Physical Review B.* **84** 10.1103/PhysRevB.84.085408 (2011).

[40] Merino, R. L. et al. Interplay between light and heavy electron bands in magic-angle twisted bilayer graphene. *Nat. Phys.* **21**, 1078–1084 (2025).

[41] Eisenstein, J. P., Pfeiffer, L. N. & West, K. W. Negative compressibility of interacting two-dimensional electron and quasiparticle gases. *Phys. Rev. Lett.* **68**, 674–677 (1992). 10.1103/PhysRevLett.68.674 10045961

[42] Bultinck, N. et al. Ground state and hidden symmetry of magic-angle graphene at even integer filling. *Phys. Rev. X* **10**, 031034 (2020).

[43] Bernevig, B. A. et al. Twisted bilayer graphene. V. Exact analytic many-body excitations in Coulomb Hamiltonians: Charge gap, Goldstone modes, and absence of Cooper pairing. *Phys. Rev. B* **103**, 205415 (2021).

[44] Rai, G. et al. Dynamical correlations and order in magic-angle twisted bilayer graphene. *Phys. Rev. X* **14**, 031045 (2024).

[45] Hu, Q. et al. Link between cascade phenomenon and correlated Chern insulators in magic-angle twisted bilayer graphene. *Nat. Commun.* **16**, 9516 (2025). 10.1038/s41467-025-64520-w PMC12569382 41152251

[46] Călugăru, D. et al. Twisted bilayer graphene as topological heavy fermion: II. Analytical approximations of the model parameters. *Low. Temp. Phys.* **49**, 640–654 (2023).

[47] Călugăru, D. et al. *Thermoelectric Effect and Its Natural Heavy Fermion Explanation in Twisted Bilayer and Trilayer Graphene*. arXiv: 2402.14057 (2024).

[48] Yu, J. C. et al. Spin skyrmion gaps as signatures of strong-coupling insulators in magic-angle twisted bilayer graphene. *Nat. Commun.* **14**, 6679 (2023). 10.1038/s41467-023-42275-6 PMC10590429 37865663

[49] Kwan, Y. H. et al. Kekul\‘e Spiral Order at All Nonzero Integer Fillings in Twisted Bilayer Graphene. *Phys. Rev. X* **11**, 041063 (2021).

[50] Nuckolls, K. P. et al. Quantum textures of the many-body wavefunctions in magic-angle graphene. *Nature* **620**, 525–532 (2023). 10.1038/s41586-023-06226-x 37587297

[51] Kim, K. et al. van der Waals Heterostructures with High Accuracy Rotational Alignment. *Nano Lett.* **16**, 1989–1995 (2016). 10.1021/acs.nanolett.5b05263 26859527

[52] Wang, L. et al. One-dimensional electrical contact to a two-dimensional material. *Science* **342**, 614–617 (2013). 10.1126/science.1244358 24179223

[53] Charlier, J. C., Gonze, X. & Michenaud, J. P. First-principles study of the electronic properties of graphite. *Phys. Rev. B* **43**, 4579–4589 (1991). 10.1103/physrevb.43.4579 9997825

[54] Partoens, B. & Peeters, F. M. From graphene to graphite: Electronic structure around the *K* point. *Phys. Rev. B* **74**, 075404 (2006).

[55] Gerischer, H. et al. Density of the electronic states of graphite: derivation from differential capacitance measurements. *J. Phys. Chem.* **91**, 1930–1935 (1987).

[56] Jung, S. et al. Direct probing of the electronic structures of single-layer and bilayer graphene with a hexagonal boron nitride tunneling barrier. *Nano Lett.* **17**, 206–213 (2017). 10.1021/acs.nanolett.6b03821 28005378

[57] Britnell, L. et al. Field-effect tunneling transistor based on vertical graphene heterostructures. *Science* **335**, 947–950 (2012). 10.1126/science.1218461 22300848

[58] Yang, H. et al. Graphene Barristor, A Triode Device With A Gate-controlled Schottky Barrier. *Science* **336**, 1140–1143 (2012). 10.1126/science.1220527 22604723

[59] Yu, J. C. et al. Correlated Hofstadter spectrum and flavour phase diagram in magic-angle twisted bilayer graphene. *Nat. Phys.* **18**, 825-+ (2022).

[60] Saito, Y. et al. Hofstadter subband ferromagnetism and symmetry-broken Chern insulators in twisted bilayer graphene. *Nat. Phys.* **17**, 478–481 (2021).

