# Supplementary material for: Entropy plateaus, combinatorial degeneracy, local moments, and heavy-fermion mass renormalization in magic-angle graphene
Source: Nat Commun. 2026 Aug 8;17:9785. doi: 10.1038/s41467-026-76179-y (PMC13575161; doi:10.1038/s41467-026-76179-y)
Supplement: Supplementary file 1 [file 41467_2026_76179_MOESM1_ESM.pdf]

Supplementary Information

Entropy Plateaus, Combinatorial Degeneracy, Local Moments, and Heavy-Fermion Mass Renormalization in Magic-Angle Graphene

Zhenyuan Zhang,[1,*] Shuang Wu,[1,*] Dumitru Călugăru,[2,3] Haoyu Hu,[2,4] Takashi Taniguchi,[5] Kenji Watanabe,[6] B. Andrei Bernevig,[2,4,7] and Eva Y. Andrei[1,†]

[1] *Department of Physics and Astronomy, Rutgers University, Piscataway, New Jersey, 08854, USA*
[2] *Department of Physics, Princeton University, Princeton, New Jersey, 08544, USA*
[3] *Rudolf Peierls Centre for Theoretical Physics, University of Oxford, Oxford OX1 3PU, United Kingdom*
[4] *Donostia International Physics Center (DIPC), Paseo Manuel de Lardizábal, 20018, San Sebastián, Spain*
[5] *International Center for Materials Nanoarchitectonics, National Institute for Materials Science, 1-1 Namiki, Tsukuba 305-0044, Japan*
[6] *Research Center for Functional Materials, National Institute for Materials Science, 1-1 Namiki, Tsukuba 305-0044, Japan*
[7] *IKERBASQUE, Basque Foundation for Science, 48013 Bilbao, Spain*

Appendix A: Methods

FIG.S1. dI_n/dV_b map and Extraction of inverse compressibility.

FIG.S2. Filling-dependent inverse compressibility with and without magnetic field.

FIG.S3. Landau levels of flat bands and remote bands.

FIG.S4. Partial filling and entropy S.

FIG.S5. Extraction of entropy.

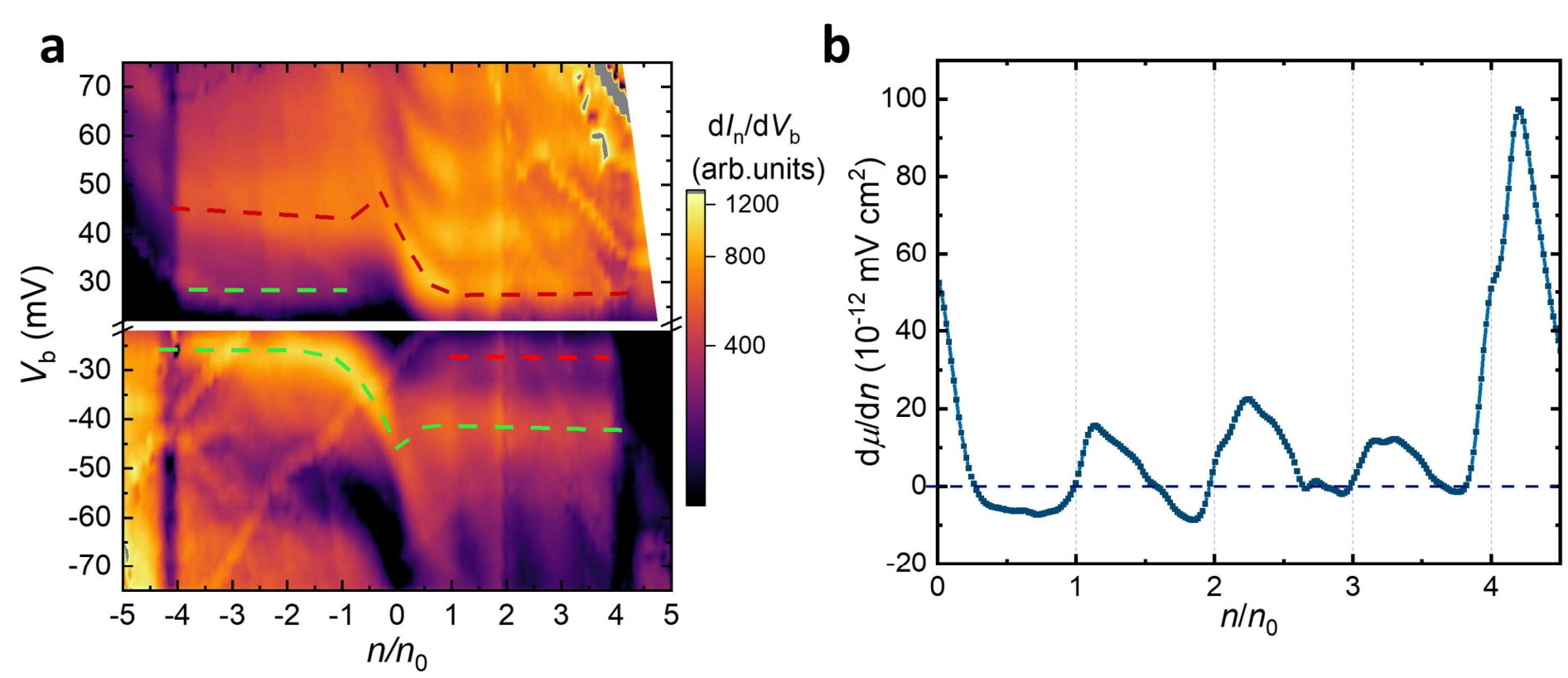

FIG. S1. **$\mathbf{dI_n/dV_b}$ map and Extraction of inverse compressibility. a,** dI_n/dV_b map as a function of V_b and n/n_0. The phonon-induced gap feature near $V_b = 0$mV is omitted. **b,** Extracted $d\mu/dn$ on the electron side at 4K with data in Fig.1e.

* These authors contributed equally to this work.
† Corresponding author.Email: andrei@physics.rutgers.edu

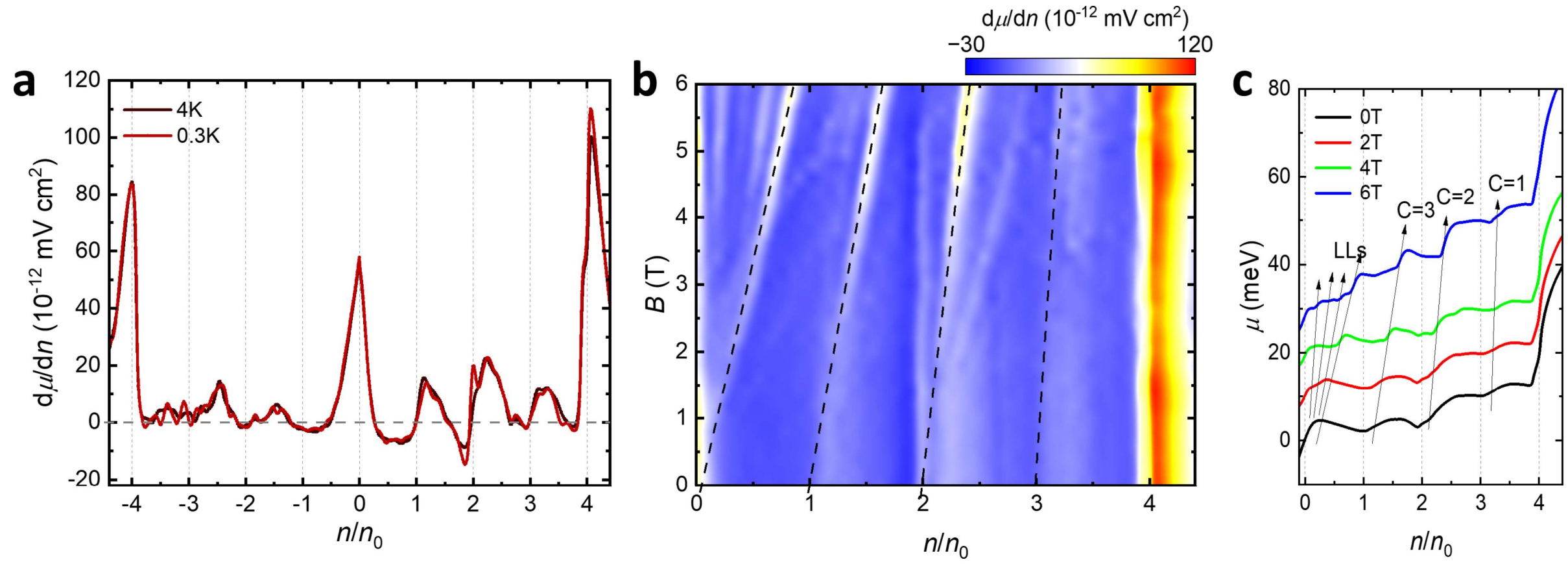

FIG. S2. **Filling-dependent inverse compressibility with and without magnetic field.** **a,** Extracted $d\mu/dn$ at 0.3K and 4K from dI/dV_b and dI/dV_g at $V_b = 42$mV **b,** $d\mu/dn$ map on the electron side as a function of filling and perpendicular magnetic field. The Landau filling factor and Chern number can be extracted from the response of $d\mu/dn$ peaks of LL and Chern gaps labeled by dashed lines. **c**, Chemical potential (stacked) on the electron side at various perpendicular magnetic fields. Arrows mark the LL and Chern gaps developing under magnetic fields. All the degeneracies of the 0th LL are lifted under high magnetic fields.

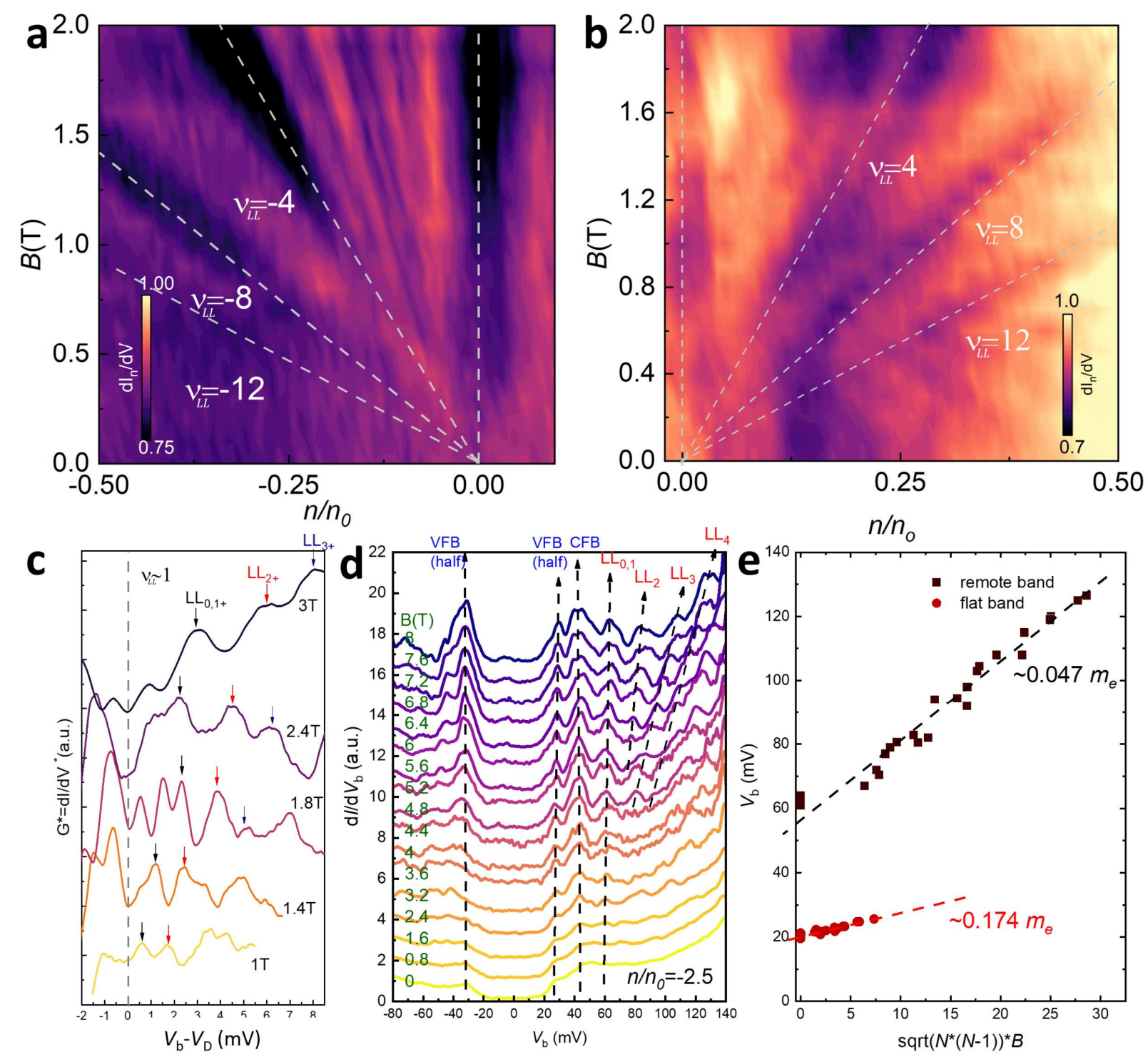

FIG. S3. **Landau levels of flat bands and remote bands a,** High-resolution dI_n/dV_b(40mV) near CNP on the hole side. The low dI_n/dV_b dark lines correspond to the high compressibility of gaps between LLs. **b,** Same as **a**, but measured at -40mV and on the electron side. **c**, dI/dV_b^*(stacked) as a function of $V_b - V_D$ (V_D is the bias voltage of the Dirac point) at the fixed $\nu_{LL} \sim 1$ near the CNP from 1 to 3T. The LLs of the flat bands are labeled with arrows. **d**, dI/dV_b (stacked) as a function of V_b at $n/n_0 = -2.5$ from 0 to 8T. The flat bands (VFB: valence flat band, CFB: conduction flat band) and the LLs of remote bands are labeled with dashed lines. e, V_b of the LL peaks as a function of $\sqrt{N(N-1)}B$ taken from **c** and **d**. $E_N - E_0 = \hbar\omega_c\sqrt{N(N-1)}$ for massive chiral bands, $\omega_c = eB/m^*$. The linear fittings claim an effective mass of $0.047m_e$ and $0.174m_e$ for remote bands and flat bands, respectively.

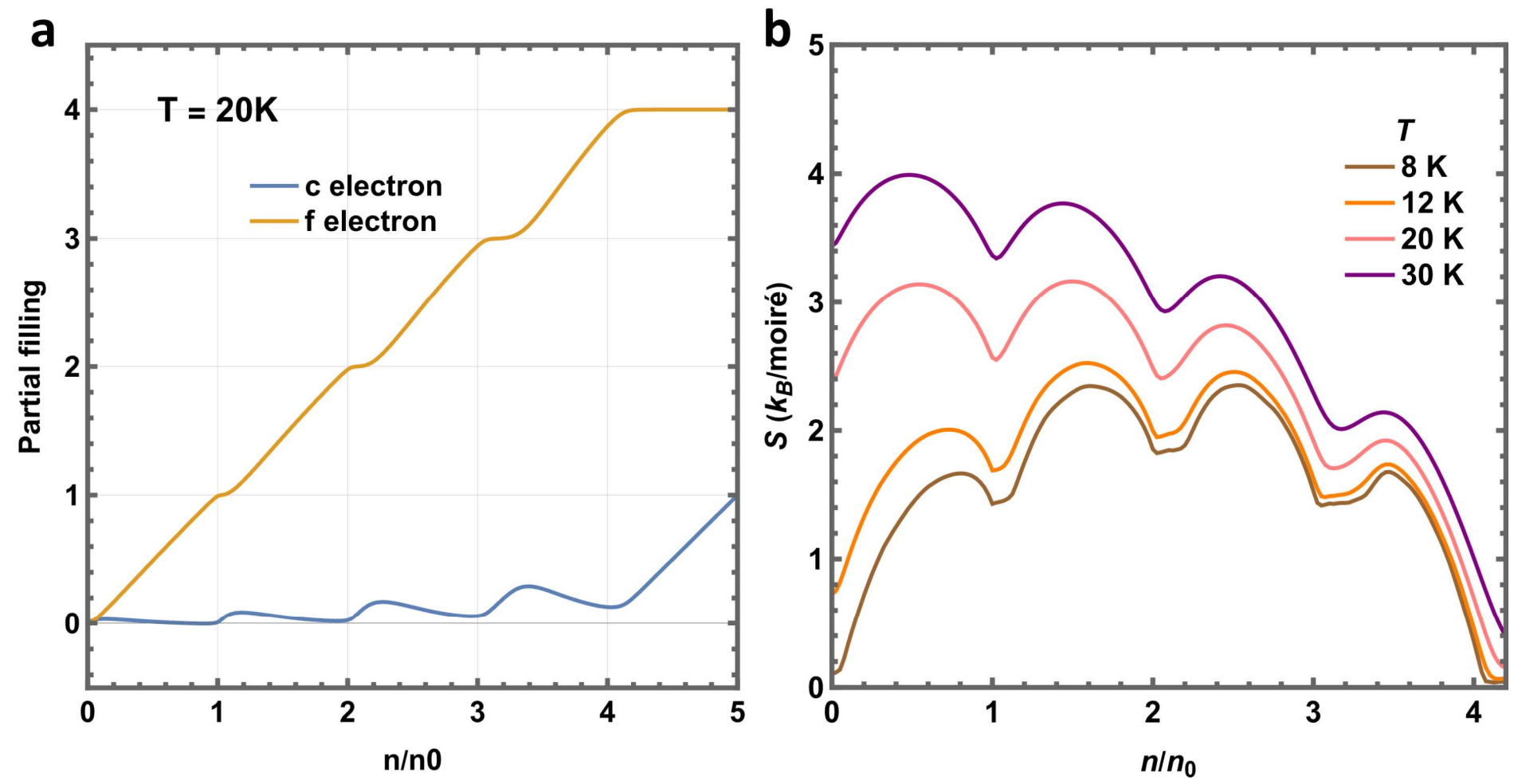

FIG. S4. **Partial filling and entropy S. a,** Partial filling of f- and c-electrons calculated in the atomic limit of the topological heavy-fermion model with strain. The Hubbard interaction energy U_1 of 23mV and the compressive heterostrain $\epsilon = 0.001$ are set to match the experimental data (Appendix I). Within the flat bands, most electrons are localized f-electrons at AA sites. Above full filling, mobile c-electrons have high occupancy in the remote bands. **b,** Total entropy S on the electron side at various temperatures calculated with the same parameters as **a**. At low temperatures below 10K, the non-interacting conduction and valence flat bands separate due to heterostrain, each with degeneracy of 4. As temperature goes up, charge transfer becomes possible between flat bands and the degeneracy gets doubled to 8, increasing the total entropy.

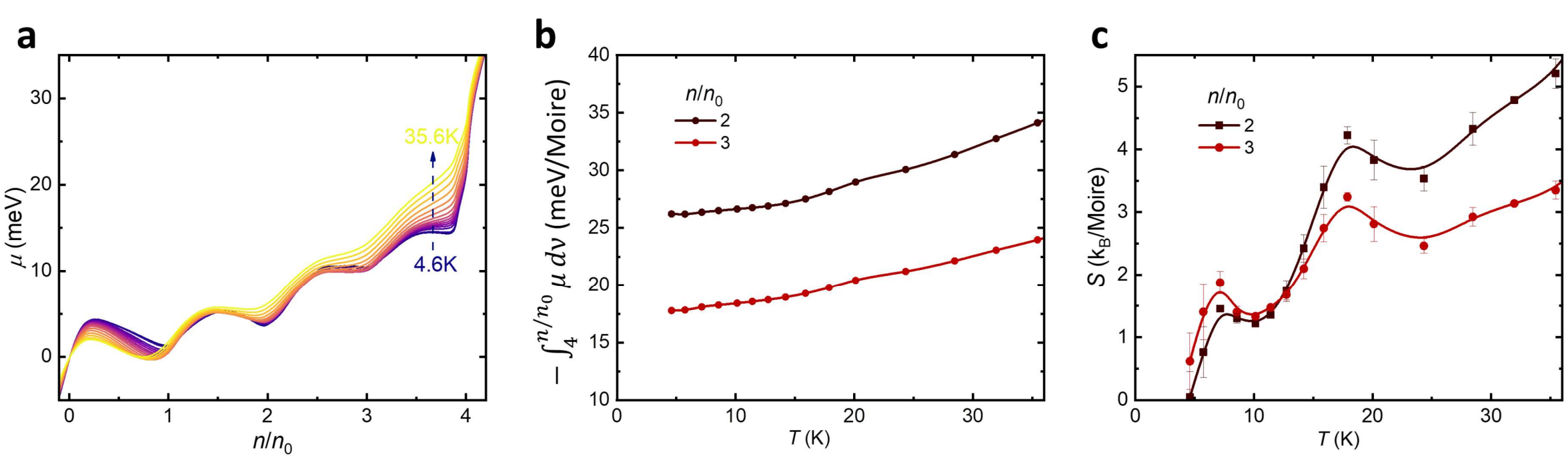

FIG. S5. **Extraction of entropy. a,** Chemical potential on the electron side from 4K to 40K. The chemical potential at CNP is set to 0. **b,** The integrals of chemical potential at filling of 2 and 3 as a function of temperature. **c**, Extracted entropy with the formula $S(\frac{n}{n_0}, T) = -\frac{\partial \int_4^{n/n_0} \mu_{n/n_0} \mathrm{d}(\frac{n}{n_0})}{\partial T}$ and data in **b**. The entropy curves show two plateaus around 10K and 20K. The error bars represent the estimated 1-sigma (1σ) uncertainty of the fitted entropy.

Appendix B: Determination of the lattice misalignment

The TBG and graphite underneath the hBN could be imaged through electrostatic force microscopy. [1] The lattice misalignment between graphite probe and TBG could be measured based on their edge orientations as shown in Fig. S6. Assuming their edge types are the same, the angle between the edge orientations of 102° (or 78°) indicates that the lattice misalignment is 18°.

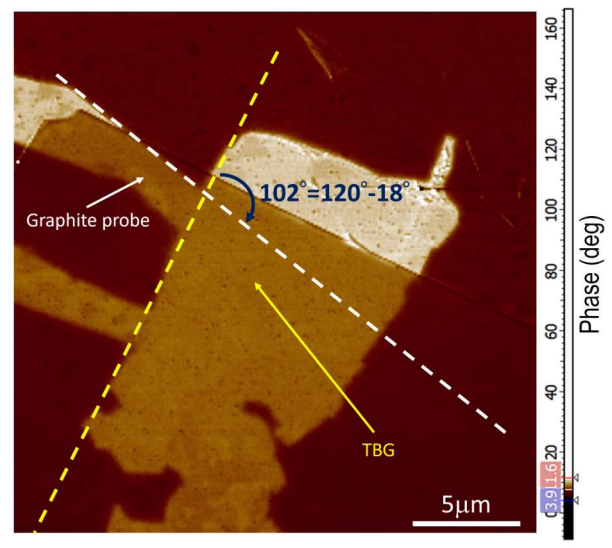

FIG. S6. **Lattice misalignment by EFM imaging.** The yellow (white) dashed line in the phase image of EFM mode indicates the edge of TBG (graphite probe). Darker contrast in TBG or graphite originates from the cover of the top hBN.

Appendix C: Fitting of transmission coefficient background

This transmission coefficient background is due to the gate dependence of the tunneling barrier height [2–4]. In our sample, the tunneling layer is a 6-layer h-BN, which is thick enough to observe the exponential $T(n)$. Through the normalization by the transmission coefficient background, the effect of tunneling rate to the chemical potential could be excluded leaving the chemical potential only depending on the filling.

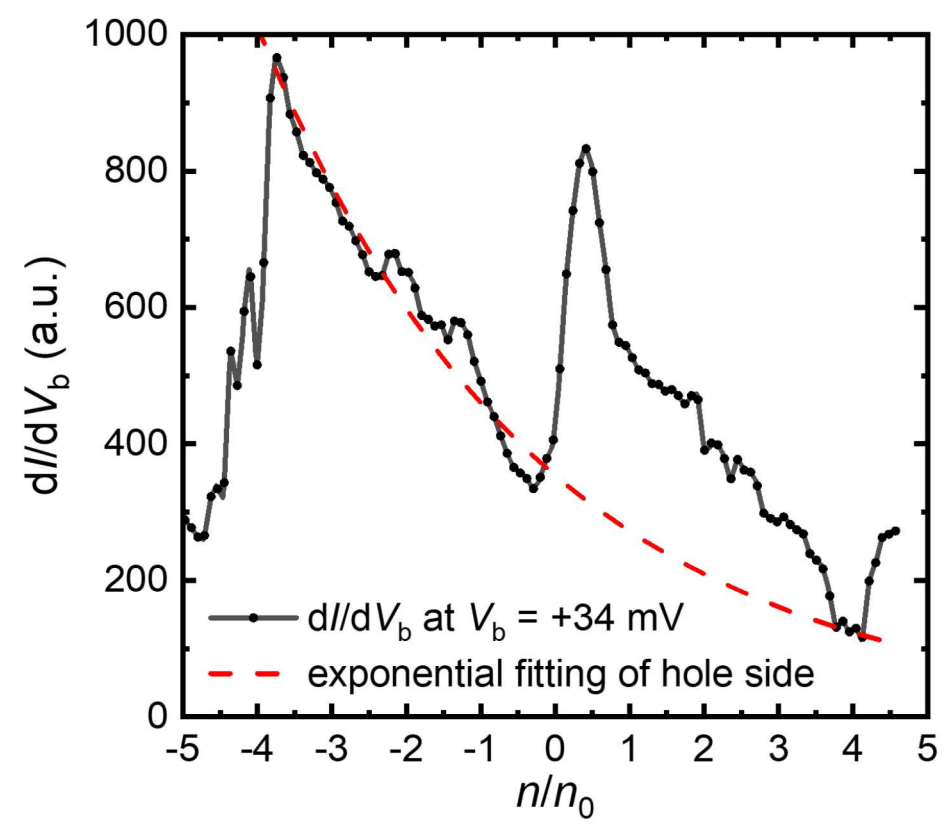

FIG. S7. **Fitting of $T(n)$ background.** $\mathrm{d}I/\mathrm{d}V_b$ curve at $V_b = 34$mV. The red dashed line is an exponential fitting of the hole side with $\mathrm{d}I/\mathrm{d}V_b = \mathrm{const} \cdot \exp(k \cdot n/n_0)$, here $k = 0.258$. The exponential term is from the filling dependence of transmission coefficient.

Appendix D: Filling correction near filling of -4 and 4

Usually, carrier density n is calculated simply using a parallel capacitor model and carrier density is proportional to the gate voltage applied. However, it is noted that using this method, the $\mathrm{d}\mu/\mathrm{d}n$ peaks (Fig.S8a) around fillings -4 and 4 are away from integer by about 0.2. This may be explained by considering quantum capacitance and geometric capacitance in series as shown below.

$$\frac{1}{C} = \frac{1}{C_q} + \frac{1}{C_g} \tag{D1}$$

Geometric capacitance C_g depends on sample-gate distance and is a known constant. Quantum capacitance C_q is proportional to the density of states. When the sample enters insulating states at bandedges, the effect of quantum capacitance becomes stronger, and the true filling needs adjustment. With the extraction of $\mathrm{d}\mu/\mathrm{d}n$ and the relationship that $C_q = e^2 \frac{\mathrm{d}n}{\mathrm{d}\mu}$, the total capaticance is calculated (Fig.S8b) and the filling is corrected (Fig.S8c). After correction, the $\mathrm{d}\mu/\mathrm{d}n$ peaks appear almost exactly at -4 and 4 (Fig.S8a). All the fillings in the paper are corrected with this method. Note that it has little effect on the filling determination within the flat bands.

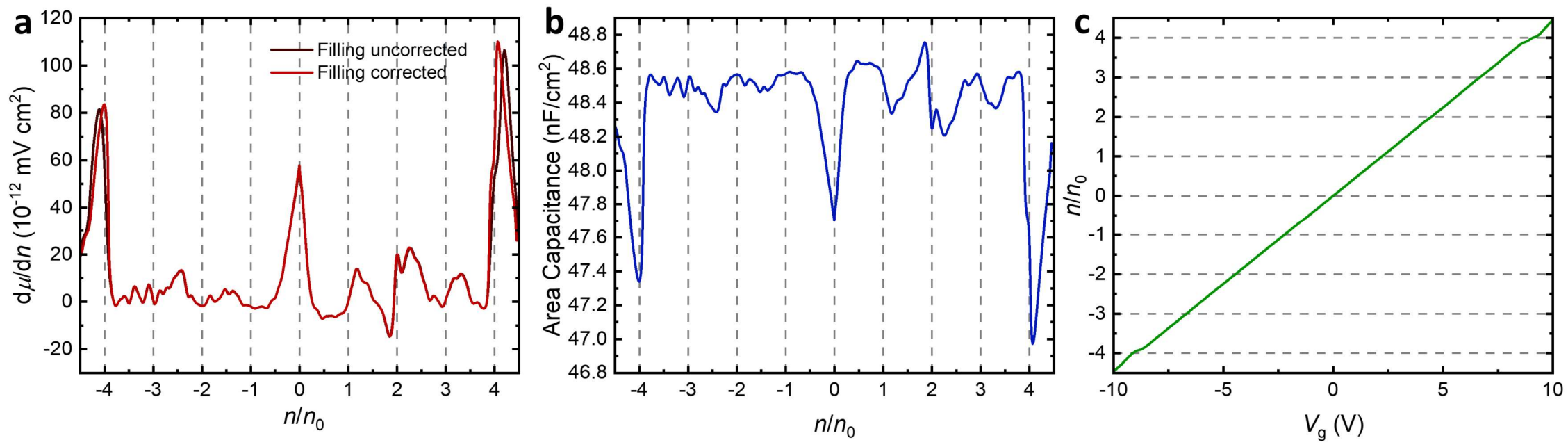

FIG. S8. **Filling correction.** **a**, $\mathrm{d}\mu/\mathrm{d}n$ before and after correction at 0.3 K. **b**, Total area capacitance combining C_g and C_q. **c**, Filling as a function of Vg. Filling is proportional to V_g within the flat bands.

Appendix E: Landau levels and Chern insulators at 6T

The Landau levels (LLs) arising from quantum Hall states of the flat bands are quite hard to observe owing to the narrow bandwidth and large effective mass. By contrast, the remote bands, which are wide and parabolic, are expected to develop LLs similar to those in Bernal bilayer graphene where the carriers are massive chiral fermions. At 6T, as the magic angle TBG sample is doped below $n/n_0 = -4$, the unoccupied bands jump away from the Fermi level at Landau filling of $\nu_{LL} = -4$ and $\nu_{LL} = -8$ marked in grey color in Fig.S9a, indicating LLs with degeneracy of 4 and Berry phase of 0 or 2π. The energy jumps below $n/n_0 = -4$ marked by purple dashed lines in Fig.S9a at Landau filling $\nu_{LL} = -4$ ($V_g^* = -11$V) and $\nu_{LL} = -8$ ($V_g^* = -13$V) are equal to the energy spacings between the corresponding remote LLs, which are comparable to the bandwidth of flat bands. The gate voltages corresponding to the CNP, LLs/Chern insulators (CIs) and band edges of the flat bands are marked in Fig.S9a-S9d.

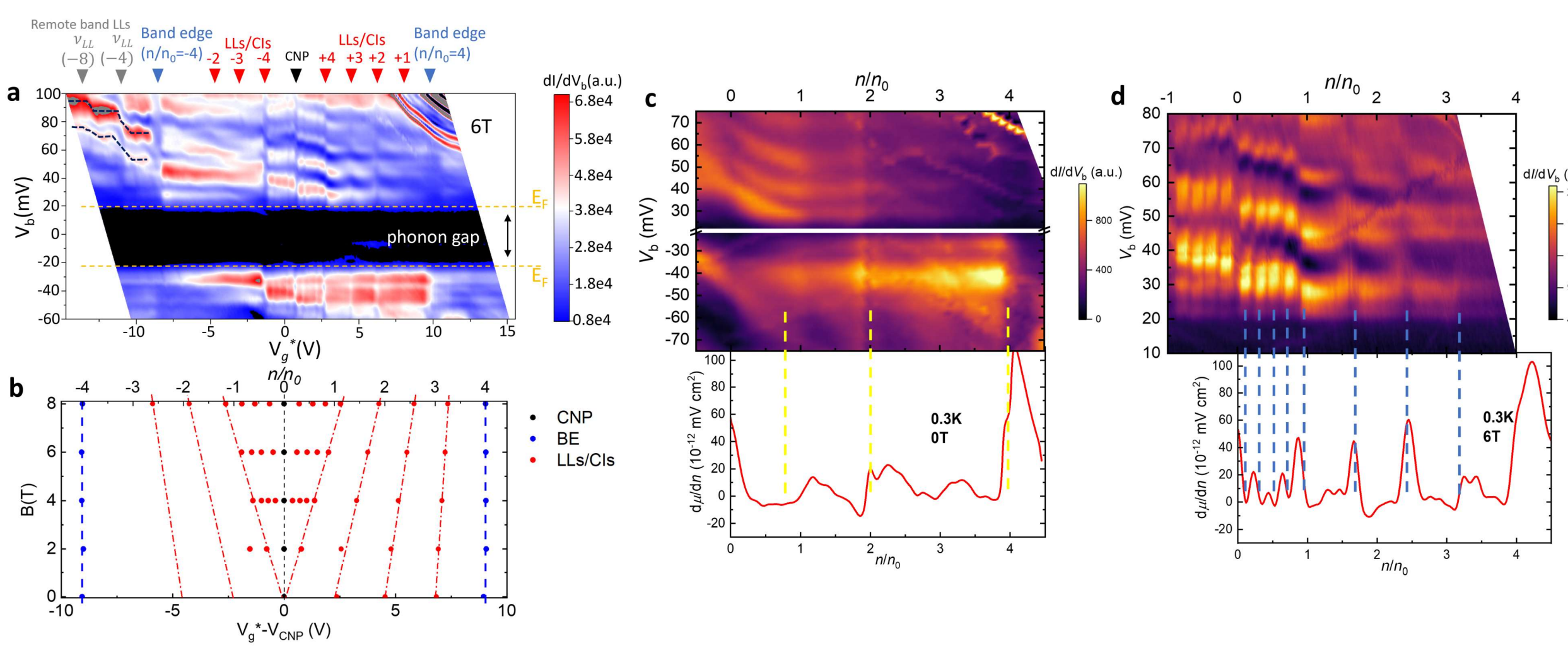

FIG. S9. **LLs and Chern insulators in TBG.** **a**, $\mathrm{d}I/\mathrm{d}V_b$ map at 6T. Gate voltages of dark vertical lines are marked with distinct colors. At high energies above 40mV, discrete flat bands are observed which are LLs of the remote bands. Flat bands below $n/n_0 = -4$ are marked by purple dashed lines. **b**, Extracted gate voltages corresponding to CNP, LLs/CIs, and band edges (BE) at various magnetic fields.**c**, Top panel: dI/dV_b map at 0.3K and zero magnetic field. Bottom: $\mathrm{d}\mu/\mathrm{d}n$ at 0.3K and zero magnetic field. The $\mathrm{d}\mu/\mathrm{d}n$ peaks at $n/n_0 = 0, 2, 4$ align well with dark vertical lines in the top panel indicating low DOS. **d**, Same as **c**, but under a perpendicular magnetic field of 6T. Peaks in $\mathrm{d}\mu/\mathrm{d}n$ caused by LLs and CIs align with dark vertical lines in the $\mathrm{d}I/\mathrm{d}V_b$ map.

Appendix F: Magnetic field dependence of dμ/dn and dI/dV$_\mathrm{b}$.

The perpendicular magnetic field dependence of dμ/dn on the hole side is shown in Fig.S10a. No prominent CIs are observed below 6T (also not observed in the dI/dV_b map at 6T in Fig. S9a). The dI/d$V_b(n/n_0, B)$ at the Fermi energy (22 mV in this case) also shows features of LLs and CIs (Fig.S10b). CIs with $C = -2, -3$ develop only above 6.5T, much weaker than on the electron side. The same trend implies a connection between cascade of transitions at zero magnetic field and the CIs at high fields (both being stronger on the electron side, both happening near integer fillings).

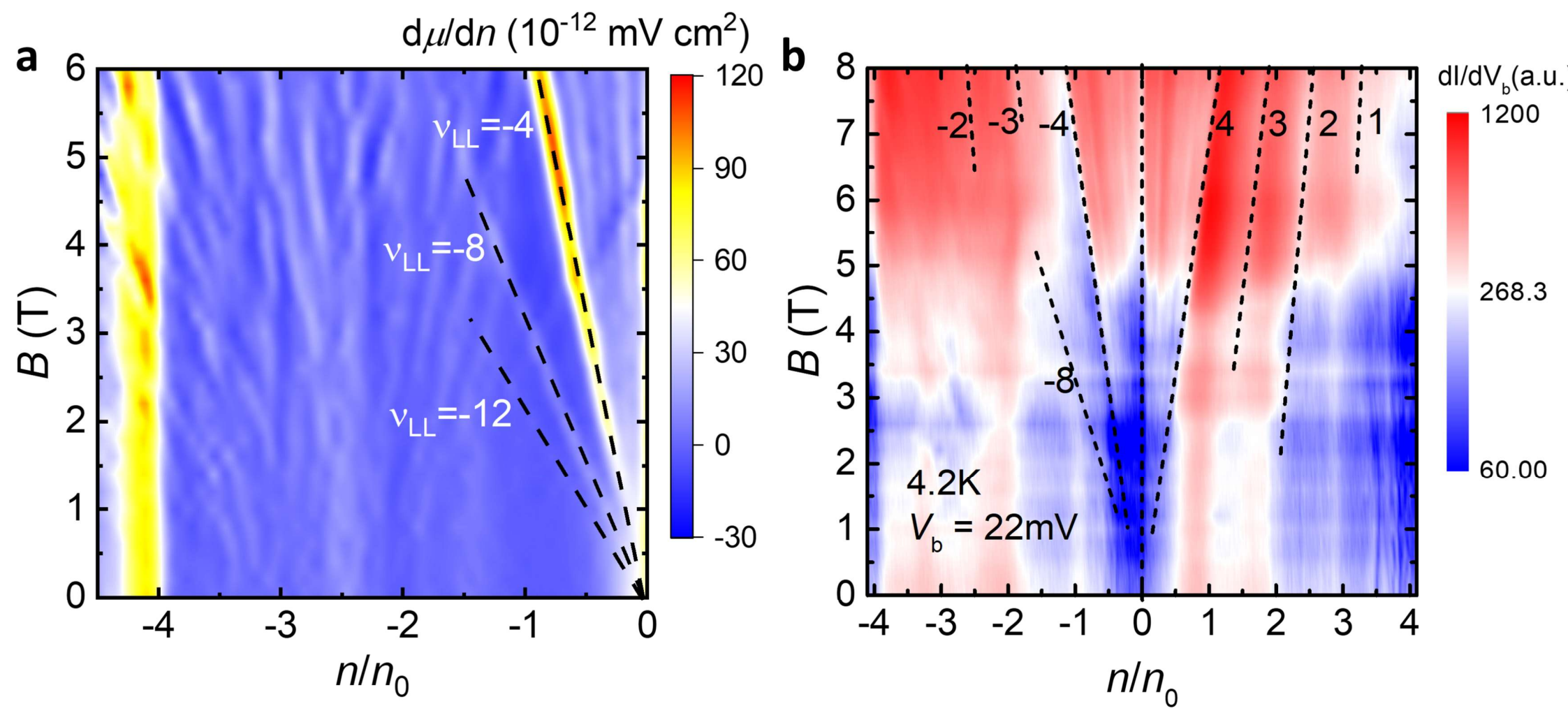

FIG. S10. **Magnetic field dependence of dμ/dn and dI/dV$_\mathrm{b}$.** **a**, $d\mu/dn$ map on the hole side as a function of filling and perpendicular magnetic field. **b**, Magnetic field dependence of dI/dV_b at the Fermi energy. CIs on the hole side develop only above 6.5 T. LLs/CIs and CNP are marked by black dashed lines.

Appendix G: The topological heavy-fermion model

1. Model

In this work, we employ the topological heavy-fermion (THF) model [5–20] to study twisted bilayer graphene (TBG). We adopt the parameters from Ref. [5] for a twist angle $\theta = 1.05°$ and a tunneling amplitude ratio $w_0/w_1 = 0.8$, incorporating strain and relaxation effects following Ref. [18].

At the single-layer graphene scale, uniform strain induces the transformation $\mathbf{r} \to (1 + \mathcal{E})\,\mathbf{r}$, where $\mathcal{E}$ is a general real two-dimensional square matrix. For each TBG layer l (with $l = +$ for the top layer and $l = -$ for the bottom), the strain tensor is given by [18]

$$\mathcal{E}^l \equiv \begin{pmatrix} \epsilon^l_+ + \epsilon^l_- & \epsilon^l_{xy} \\ \epsilon^l_{xy} & \epsilon^l_+ - \epsilon^l_- \end{pmatrix}, \tag{G1}$$

where ϵ^l_+ and ϵ^l_- denote the isotropic and anisotropic strain components, respectively, and ϵ^l_{xy} represents the shear strain. These strain tensors can be decomposed into symmetric (homostrain, $\mathcal{E}^- + \mathcal{E}^+$) and antisymmetric (heterostrain, $\mathcal{E}^- - \mathcal{E}^+$) combinations. Since homostrain has negligible effects on TBG's band structure to leading order [18], we focus solely on heterostrain, characterized by opposite strains in the two layers, *i.e.*, $\mathcal{E}^- = -\mathcal{E}^+$. Thus, we define

$$\mathcal{E} = \mathcal{E}^- - \mathcal{E}^+, \quad \epsilon_\pm = \epsilon^-_\pm - \epsilon^+_\pm, \quad \epsilon_{xy} = \epsilon^-_{xy} - \epsilon^+_{xy}, \tag{G2}$$

with typical values of $\epsilon_\pm$ and ϵ_{xy} ranging from 0.001 to 0.004. In this study, we consider compressive heterostrain ϵ along the $\hat{\mathbf{x}}$ direction of the conventional TBG unit cell [5, 19], where $\epsilon_{xy} = 0$ and $\epsilon_\mp = -(1 \pm \nu_G)\,\epsilon/2$, with $\nu_G = 0.16$ being graphene's Poisson ratio [19]. Throughout, ϵ refers to compressive heterostrain along the $\hat{\mathbf{x}}$ axis.

We adjust the interaction parameters from Ref. [5] to align with the experimental geometry considered here. Specifically, we model the Coulomb interaction between electrons using a single-gate screened potential [21]

$$V(\mathbf{q}) = 2\pi U_\xi \xi^2 \frac{1-e^{-2\xi q}}{\xi q}, \tag{G3}$$

where $U_\xi = \frac{e^2}{4\pi\varepsilon_0\varepsilon\xi}$ sets the interaction energy scale, ε is the dielectric constant, and ξ denotes the distance between the sample and the screening gate. We use $\varepsilon = 12$ [22] and $\xi = 2\,\mathrm{nm}$, resulting in $U_\xi = 60\,\mathrm{meV}$. The THF interaction parameters are then [5]

$$\begin{aligned} U_1 &= 23.095\,\mathrm{meV}, & U_2 &= 0.77\,\mathrm{meV}, & W_1 = W_2 &= 17.41\,\mathrm{meV},\\ J &= 6.965\,\mathrm{meV}, & K &= 2.17\,\mathrm{meV}, & W_3 = W_4 &= 20.12\,\mathrm{meV}. \end{aligned} \tag{G4}$$

The meaning of these parameters is the same as in Ref. [5]: U_1 and U_2 denote, respectively, the onsite and nearest-neighbor f-electron repulsion; the parameters W_a (for $1 \leq a \leq 4$) quantify the Coulomb repulsion between f- and c-electrons; J describes the ferromagnetic exchange between f- and c-electrons and the double hybridization interaction, while K corresponds to the density hybridization interaction. The c-c interaction potential follows the same form as in Eq. (G3).

2. Hartree-Fock simulations in the symmetry-broken phase

We perform simulations incorporating strain and relaxation effects to compute the compressibility of the incommensurate Kekulé spiral (IKS) ground state near $\nu = +2$. To this end, we first obtain the self-consistent Hartree-Fock solution of the IKS state exactly at integer filling $\nu = +2$, utilizing the analytical variational ansatz for the corresponding density matrix derived in Ref. [19]. We apply a strain of $\varepsilon = 0.001$ in these calculations.

Once the self-consistent solution at integer filling is obtained, we determine the Hartree-Fock self-consistent density matrix for non-integer fillings by incrementally doping the system. To ensure efficient convergence, we employ standard acceleration algorithms [23–27]. At fractional fillings, where the system becomes gapless, convergence is further improved by introducing a small but finite temperature.

From the Hartree-Fock simulations, we can extract the dependence of the chemical potential on the filling, $\mu(\nu)$. To enable a direct comparison with experimental results, we subtract the classical charging energy associated with uniformly charging the TBG sample to a filling ν. This procedure effectively removes a linear contribution in ν from the chemical potential [17], leading to the following adjustment

$$\mu \to \mu - \frac{1}{\Omega_0} V(\mathbf{0})\,\nu. \tag{G5}$$

Appendix H: The atomic limit of the topological heavy-fermion model with strain

In this section, we consider the entropy of twisted bilayer graphene (TBG) within the topological heavy-fermion (THF) model in atomic limit. In the atomic limit, the f-electrons are treated as uncoupled from the c-electrons at either the single-particle or interaction level. This allows us to analytically obtain the entropy of f-electrons in the presence of strain effects.

1. Atomic limit Hamiltonian

The atomic limit for the THF model of TBG [5] was considered by Ref. [28]. In the atomic limit, the f-electrons at different lattice sites are decoupled from one another and also decoupled from the c-electrons. Because in this limit, the f-electrons at a given lattice site are not hybridizing or interacting with any other fermions, we can study the atomic problem via the following grand canonical Hamiltonian,

$$K^{\mathrm{At}} = \sum_{\substack{\alpha,\beta\\ \eta,s}} \left(M_f \left[\epsilon_{xy}\sigma_x + \eta\epsilon_-\sigma_y\right]_{\alpha\beta} - \mu\delta_{\alpha\beta} \right) \hat{f}^\dagger_{\alpha,\eta,s}\hat{f}_{\beta,\eta,s} + \frac{U_1}{2} \sum_{\substack{\alpha,\eta,s\\ \alpha',\eta',s'}} \left(\hat{f}^\dagger_{\alpha,\eta,s}\hat{f}_{\alpha,\eta,s} - \frac{1}{2} \right) \left(\hat{f}^\dagger_{\alpha',\eta',s'}\hat{f}_{\alpha',\eta',s'} - \frac{1}{2} \right). \tag{H1}$$

In Eq. (H1), $\hat{f}^\dagger_{\alpha,\eta,s}$ creates an f-electron at a given lattice site $\mathbf{R}_0$ (since the system is translationally invariant, the exact choice of $\mathbf{R}_0$ is unimportant) in valley $\eta = \pm$, orbital $\alpha = 1, 2$, and having spin $s = \uparrow, \downarrow$. The first term in Eq. (H1) denotes f-electron strain contribution to the single-particle dispersion of the THF model, as derived by Ref. [18], where $M_f = 4252\,\text{meV}$ and σ_x, σ_y denoting the Pauli matrices acting on the f-electron orbital subspace. The second term of Eq. (H1) is just the $\mathrm{U}(8)$ onsite Hubbard repulsion of the THF model, with U_1 denoting the corresponding interacting energy scale [5]. The zero-strain limit of Eq. (H1) was extensively studied in Ref. [28].

In what follows, we will find it useful to work in the basis in which single-particle term of K^{At} is diagonal. To this end, we define *rotated* f-fermion operators $\hat{f}^\dagger_{a,\eta,s}$, where $a = \pm$, where

$$\begin{pmatrix} \hat{f}^\dagger_{+,\eta,s} \\ \hat{f}^\dagger_{-,\eta,s} \end{pmatrix} = \frac{1}{\sqrt{2}} \begin{pmatrix} 1 & \frac{\epsilon_{xy}+i\eta\epsilon_-}{\sqrt{\epsilon_{xy}^2+\epsilon_-^2}} \\ 1 & -\frac{\epsilon_{xy}+i\eta\epsilon_-}{\sqrt{\epsilon_{xy}^2+\epsilon_-^2}} \end{pmatrix} \begin{pmatrix} \hat{f}^\dagger_{1,\eta,s} \\ \hat{f}^\dagger_{2,\eta,s} \end{pmatrix}. \tag{H2}$$

We then define the number operators

$$\hat{n}_a = \sum_{\eta,s} \hat{f}^\dagger_{a,\eta,s} \hat{f}_{a,\eta,s}, \tag{H3}$$

in terms of which the atomic-limit grand canonical Hamiltonian is given by

$$K^{\text{At}} = \sum_{a=\pm} (a\varepsilon - \mu)\,\hat{n}_a + \frac{U_1}{2}\left(\sum_{a=\pm} \hat{n}_a - \frac{N_f}{2}\right)^2, \tag{H4}$$

where $N_f = 8$ denotes the number of f-fermion flavors, while $\varepsilon = |M_f|\sqrt{\epsilon_{xy}^2 + \epsilon_-^2}$ is half the f-electron strain-splitting.

2. Entropy in the atomic limit

The single-site partition function corresponding to the atomic limit Hamiltonian from Eq. (H4) is given by

$$\begin{aligned} Z^{\text{At}}(\beta,\mu) &\equiv \operatorname{Tr}\left(e^{-\beta K^{\text{At}}}\right) \\ &= \sum_{n_+,n_-=0}^{N_f/2} \binom{\frac{N_f}{2}}{n_-}\binom{\frac{N_f}{2}}{n_+} e^{-\beta\left[\varepsilon(n_+-n_-)-\mu(n_++n_-)+\frac{U_1}{2}\left(n_++n_--\frac{N_f}{2}\right)^2\right]} \\ &= \sum_{N=-\frac{N_f}{2}}^{\frac{N_f}{2}} \sum_{n=\frac{|N|}{2}-\frac{N_f}{4}}^{\frac{N_f}{4}-\frac{|N|}{2}} \binom{\frac{N_f}{2}}{\frac{N}{2}-n+\frac{N_f}{4}}\binom{\frac{N_f}{2}}{\frac{N}{2}+n+\frac{N_f}{4}} e^{-\beta\left[2\varepsilon n-\mu\left(N+\frac{N_f}{2}\right)+\frac{U_1}{2}N^2\right]}, \end{aligned} \tag{H5}$$

where $\beta = \frac{1}{T}$ is the inverse temperature. By definition, the corresponding single-site grand potential is

$$\Phi^{\text{At}}(\beta,\mu) = -\frac{1}{\beta}\log Z^{\text{At}}(\beta,\mu), \tag{H6}$$

from which the entropy can be extracted using the thermodynamic relation

$$S^{\text{At}}(\beta,\mu) = -\frac{\partial\Phi^{\text{At}}(\beta,\mu)}{\partial\left(\frac{1}{\beta}\right)} = \beta^2\frac{\partial\Phi^{\text{At}}(\beta,\mu)}{\partial\beta}. \tag{H7}$$

In principle, the partition function (and, as a result, the corresponding entropy) can be computed numerically for different values of U_1 and ε as a function of μ and β. In order to get some analytical understanding of the results, we will also get approximate analytical expressions for the entropy in two experimentally-relevant regimes: $\frac{1}{\beta} \ll \varepsilon \ll U_1$ (the low-temperature regime) and $\varepsilon \ll \frac{1}{\beta} \ll U_1$ (the high-temperature regime). Note that the strain splitting of the f-electrons is always smaller much smaller than their onsite Hubbard repulsion ($\varepsilon \ll U_1$). At the same time, for the temperatures employed in the experiments $\frac{1}{\beta} \ll U_1$ (the interaction TBG scale corresponds to hundreds of Kelvin).

To derive analytical approximations for the entropy, we start by defining the following probability distribution

$$p(N,n) = \frac{d(N,n)}{Z^{\mathrm{At}}(\beta,\mu)} e^{-\beta\left[2\varepsilon n - \mu\left(N+\frac{N_f}{2}\right) + \frac{U_1}{2}N^2\right]}, \quad \text{with} \quad d(N,n) = \binom{\frac{N_f}{2}}{\frac{N}{2} - n + \frac{N_f}{4}} \binom{\frac{N_f}{2}}{\frac{N}{2} + n + \frac{N_f}{4}}, \tag{H8}$$

such that $p(N,n)$ denotes the probability of the system having $N + N_f$ occupied f fermions, with $2n$ more fermions in occupying the $+\varepsilon$ strain band compared to the $-\varepsilon$ one, a state which has degeneracy $d(N,n)$. In terms of the probability function defined in Eq. (H8), the single-site entropy can be directly obtained from Eqs. (H6) and (H7)

$$\begin{aligned} S^{\mathrm{At}}(\beta,\mu) &= \beta^2 \frac{\partial}{\partial \beta}\left(-\frac{1}{\beta}\log Z^{\mathrm{At}}(\beta,\mu)\right) \\ &= \log Z^{\mathrm{At}}(\beta,\mu) - \frac{\beta}{Z^{\mathrm{At}}(\beta,\mu)} \frac{\partial \log Z^{\mathrm{At}}(\beta,\mu)}{\partial\beta} \\ &= -\sum_{N=-\frac{N_f}{2}}^{\frac{N_f}{2}} \sum_{n=\frac{|N|}{2}-\frac{N_f}{4}}^{\frac{N_f}{4}-\frac{|N|}{2}} p(N,n)\log\frac{p(N,n)}{d(N,n)}. \end{aligned} \tag{H9}$$

At the same time, the total f-electron filling (henceforth denoted by $-\frac{N_f}{2} \leq \nu \leq \frac{N_f}{2}$) is given by

$$\nu = \sum_{N=-\frac{N_f}{2}}^{\frac{N_f}{2}} \sum_{n=\frac{|N|}{2}-\frac{N_f}{4}}^{\frac{N_f}{4}-\frac{|N|}{2}} N p(N,n). \tag{H10}$$

a. *Asymptotic expressions for the atomic-limit entropy in the low-temperature regime*

By definition, in this limit, $1 \ll \beta\varepsilon \ll \beta U_1$. As a result, we find that

$$\frac{p(N,n_1)}{p(N,n_2)} \sim e^{-2\beta\varepsilon(n_1-n_2)} \ll 1, \tag{H11}$$

where the order-one combinatorial prefactor has been dropped, as it is less important than the exponential. In turn, this implies that in Eqs. (H9) and (H10), for every N only the terms with the smallest n give a non-vanishing contribution, which allows us to approximate

$$S^{\mathrm{At}}(\beta,\mu) \approx -\sum_{N=-\frac{N_f}{2}}^{\frac{N_f}{2}} p'(N)\log\frac{p'(N)}{d'(N)}, \tag{H12}$$

$$\nu \approx \sum_{N=-\frac{N_f}{2}}^{\frac{N_f}{2}} N p'(N), \tag{H13}$$

where, for simplicity, we have defined

$$\begin{aligned} p'(N) &= p\left(N, \frac{|N|}{2} - \frac{N_f}{4}\right) \\ &= \frac{1}{Z^{\mathrm{At}}(\beta,\mu)} \binom{\frac{N_f}{2}}{\frac{N}{2} - \frac{|N|}{2} + \frac{N_f}{2}} \binom{\frac{N_f}{2}}{\frac{N}{2} + \frac{|N|}{2}} e^{-\beta\left[\varepsilon\left(|N|-\frac{N_f}{2}\right) - \mu\left(N+\frac{N_f}{2}\right) + \frac{U_1}{2}N^2\right]} \\ &= \frac{d'(N)}{Z^{\mathrm{At}}(\beta,\mu)} e^{-\beta\left[\varepsilon\left(|N|-\frac{N_f}{2}\right) - \mu\left(N+\frac{N_f}{2}\right) + \frac{U_1}{2}N^2\right]}, \quad \text{with} \quad d'(N) = \binom{\frac{N_f}{2}}{|N|}. \end{aligned} \tag{H14}$$

Moving forward, we note that the leading behavior of $p'(N)$ with N is governed by the exponential prefactor, *i.e.*

$$p'(N) \sim e^{\beta\left(\mu N - \frac{U_1}{2}N^2\right)}, \tag{H15}$$

which implies that at a given chemical potential, there will be at most two non-vanishing probabilities: $p'(N)$ and $p'(N+1)$, for some $-\frac{N_f}{2} \leq N \leq \frac{N_f}{2} - 1$. Therefore, at a given filling ν, the only way to satisfy Eq. (H13) is if

$$p'(N) = \begin{cases} \lfloor \nu \rfloor + 1 - \nu & \text{if} \quad N = \lfloor \nu \rfloor \\ \nu - \lfloor \nu \rfloor & \text{if} \quad N = \lfloor \nu \rfloor + 1 \\ 0 & \text{otherwise} \end{cases}, \tag{H16}$$

where $\lfloor \nu \rfloor$ denotes the largest integer smaller than ν. In turn, through Eq. (H12), this allows us to obtain the low-temperature entropy as a function of ν analytically

$$S^{\text{At}}(\beta, \nu) \approx -\left(\lfloor \nu \rfloor + 1 - \nu\right) \log \frac{\lfloor \nu \rfloor + 1 - \nu}{\binom{\frac{N_f}{2}}{|\lfloor \nu \rfloor|}} - \left(\nu - \lfloor \nu \rfloor\right) \log \frac{\nu - \lfloor \nu \rfloor}{\binom{\frac{N_f}{2}}{|\lfloor \nu \rfloor + 1|}}, \quad \text{for} \quad 1 \ll \beta\varepsilon \ll \beta U_1. \tag{H17}$$

Exactly at integer fillings, Eq. (H12) reduces to

$$S^{\text{At}}(\beta, \nu) \approx \log \binom{\frac{N_f}{2}}{|\nu|}, \quad \text{for} \quad \nu \in \mathbb{Z}. \tag{H18}$$

b. *Asymptotic expressions for the atomic-limit entropy in the high-temperature regime*

In this limit, $\beta\varepsilon \ll 1 \ll \beta U_1$, as a result of which we can ignore the term proportional to ε in the exponential factors of Eq. (H5). Under this approximation, the partition function of the single-site problem reduces to its zero-strain form derived in Ref. [28],

$$Z^{\text{At}}(\beta, \mu) \approx \sum_{N=-\frac{N_f}{2}}^{\frac{N_f}{2}} \binom{N_f}{N + \frac{N_f}{2}} e^{\beta\left[\mu\left(N + \frac{N_f}{2}\right) - \frac{U_1}{2} N^2\right]}. \tag{H19}$$

Similarly to Eq. (H8), we now define the following probability distribution

$$p(N) = \frac{d(N)}{Z^{\text{At}}(\beta, \mu)} e^{\beta\left[\mu\left(N + \frac{N_f}{2}\right) - \frac{U_1}{2} N^2\right]}, \quad \text{with} \quad d(N) = \binom{N_f}{N + \frac{N_f}{2}}, \tag{H20}$$

where $p(N)$ denotes the probability of the system having $N + \frac{N_f}{2}$ electrons, while $d(N)$ denotes the total number of such states. Using Eqs. (H6) and (H7), we obtain the following expression for the entropy of the system

$$S^{\text{At}}(\beta, \mu) \approx -\sum_{N=-\frac{N_f}{2}}^{\frac{N_f}{2}} p(N) \log \frac{p(N)}{d(N)}, \tag{H21}$$

which is valid for $\beta\epsilon \ll 1$. At the same time, the filling of the system can be found directly from the partition function in Eq. (H19)

$$\nu \approx \sum_{N=-\frac{N_f}{2}}^{\frac{N_f}{2}} N p(N). \tag{H22}$$

Similarly to Eq. (H15), the leading behavior of $p(N)$ with N is again governed by the exponential prefactor

$$p(N) \sim e^{\beta\left(\mu N - \frac{U_1}{2} N^2\right)}, \tag{H23}$$

which again implies that at most two probabilities $p(N)$ (corresponding to consecutive integers N) are non-vanishing at a given chemical potential. As a result, one again finds that

$$p(N) = \begin{cases} \lfloor \nu \rfloor + 1 - \nu & \text{if} \quad N = \lfloor \nu \rfloor \\ \nu - \lfloor \nu \rfloor & \text{if} \quad N = \lfloor \nu \rfloor + 1 \\ 0 & \text{otherwise} \end{cases}, \tag{H24}$$

such that the high-temperature entropy from Eq. (H21) has the following analytical expression

$$S^{\mathrm{At}}(\beta,\nu) \approx -\left(\lfloor\nu\rfloor + 1 - \nu\right)\log\frac{\lfloor\nu\rfloor+1-\nu}{\binom{N_f}{\lfloor\nu\rfloor+\frac{N_f}{2}}} - \left(\nu - \lfloor\nu\rfloor\right)\log\frac{\nu-\lfloor\nu\rfloor}{\binom{N_f}{\lfloor\nu\rfloor+\frac{N_f}{2}+1}}, \quad \text{for} \quad 1 \ll \beta\varepsilon \ll \beta U_1. \tag{H25}$$

Exactly at integer fillings, Eq. (H12) reduces to

$$S^{\mathrm{At}}(\beta,\nu) \approx \log\binom{N_f}{\nu+\frac{N_f}{2}}, \quad \text{for} \quad \nu\in\mathbb{Z}. \tag{H26}$$

Appendix I: Zero-hybridization limit

In the atomic limit discussed in Section H, we neglected the contribution of the c-electrons to the entropy of TBG. In contrast, the zero-hybridization limit considered in this section includes the entropy contributions from both f and c-electrons while ignoring any single-particle coupling between them. Apart from the inclusion of strain [18], the zero-hybridization Hamiltonian is identical to that in Ref. [9]. Explicitly, the Hamiltonian is given by

$$H = H_c + H_{U_1} + H_V + H_W + H_{\mathrm{Strain}}, \tag{I1}$$

where H_c represents the kinetic term for the c-electrons

$$H_c = \sum_{\mathbf{k},\eta,s}\begin{pmatrix}\hat{c}^\dagger_{\mathbf{k},1,\eta,s}\\ \hat{c}^\dagger_{\mathbf{k},2,\eta,s}\\ \hat{c}^\dagger_{\mathbf{k},3,\eta,s}\\ \hat{c}^\dagger_{\mathbf{k},4,\eta,s}\end{pmatrix}^T\begin{pmatrix}\mathbb{0} & v_\star\left(\eta k_x\sigma_0 + i k_y\sigma_z\right)\\ v_*\left(\eta k_x\sigma_0 - i k_y\sigma_z\right) & M\sigma_z\end{pmatrix}\begin{pmatrix}\hat{c}_{\mathbf{k},1,\eta,s}\\ \hat{c}_{\mathbf{k},2,\eta,s}\\ \hat{c}_{\mathbf{k},3,\eta,s}\\ \hat{c}_{\mathbf{k},4,\eta,s}\end{pmatrix}. \tag{I2}$$

Here, $\mathbb{0}$ denotes the zero matrix, and the single-particle parameters $v_\star$ and M are specified in Ref. [5].

The interaction terms in Eq. (I1) include the onsite f-electron repulsion (H_{U_1}), the c-electron Coulomb interaction (H_V), and the f-c density-density interaction (H_W) [5]:

$$H_{U_1} = \frac{U_1}{2}\sum_{\mathbf{R}}\sum_{\substack{\alpha,\eta,s\\ \alpha',\eta',s'}} :\hat{f}^\dagger_{\mathbf{R},\alpha,\eta,s}\hat{f}_{\mathbf{R},\alpha,\eta,s}::\hat{f}^\dagger_{\mathbf{R},\alpha',\eta',s'}\hat{f}_{\mathbf{R},\alpha',\eta',s'}:, \tag{I3}$$

$$H_V = \frac{1}{2\Omega_0 N_0}\sum_{\mathbf{k}_1,\mathbf{k}_2}\sum_{\mathbf{q}}\sum_{\substack{a,\eta,s\\ a',\eta',s'}} V(\mathbf{q}) :\hat{c}^\dagger_{\mathbf{k}_1+\mathbf{q},a,\eta,s}\hat{c}_{\mathbf{k}_1,a,\eta,s}::\hat{c}^\dagger_{\mathbf{k}_2-\mathbf{q},a',\eta',s'}\hat{c}_{\mathbf{k}_2,a',\eta',s'}:, \tag{I4}$$

$$H_W = \frac{1}{N_0}\sum_{\mathbf{k}_1,\mathbf{k}_2}\sum_{\mathbf{q}}\sum_{\substack{\alpha,\eta,s\\ a',\eta',s'}} W_{a'} :\hat{f}^\dagger_{\mathbf{k}_1+\mathbf{q},\alpha,\eta,s}\hat{f}_{\mathbf{k}_1,\alpha,\eta,s}::\hat{c}^\dagger_{\mathbf{k}_2-\mathbf{q},a',\eta',s'}\hat{c}_{\mathbf{k}_2,a',\eta',s'}: . \tag{I5}$$

In these expressions, Ω_0 denotes the surface area of the TBG unit cell, N_0 is the number of unit cells in the sample, and $V(\mathbf{q})$, U_1, and W_a (for $1 \leq a \leq 4$) are given in Section G.

The strain effects are captured by H_{Strain}, defined as [18]

$$H_{\mathrm{Strain}} = \sum_{\mathbf{k},\eta,s}\left[\begin{pmatrix}\hat{c}^\dagger_{\mathbf{k},1,\eta,s}\\ \hat{c}^\dagger_{\mathbf{k},2,\eta,s}\\ \hat{c}^\dagger_{\mathbf{k},3,\eta,s}\\ \hat{c}^\dagger_{\mathbf{k},4,\eta,s}\end{pmatrix}^T\begin{pmatrix} c\left(\epsilon_{xy}\sigma_x + \eta\epsilon_-\sigma_y\right) & c'\left(\epsilon_{xy}\sigma_x - \eta\epsilon_-\sigma_y\right)\\ c'\left(\epsilon_{xy}\sigma_x + \eta\epsilon_-\sigma_y\right) & M'\epsilon_+\sigma_2\end{pmatrix}\begin{pmatrix}\hat{c}_{\mathbf{k},1,\eta,s}\\ \hat{c}_{\mathbf{k},2,\eta,s}\\ \hat{c}_{\mathbf{k},3,\eta,s}\\ \hat{c}_{\mathbf{k},4,\eta,s}\end{pmatrix}\right.$$
$$\left. + \begin{pmatrix}\hat{f}^\dagger_{\mathbf{k},1,\eta,s}\\ \hat{f}^\dagger_{\mathbf{k},2,\eta,s}\end{pmatrix}^T M_f\left(\epsilon_{xy}\sigma_x + \eta\epsilon_-\sigma_y\right)\begin{pmatrix}\hat{f}_{\mathbf{k},1,\eta,s}\\ \hat{f}_{\mathbf{k},2,\eta,s}\end{pmatrix}\right]. \tag{I6}$$

The strain parameters c, c', M', and M_f are provided in Ref. [18].

In the zero-hybridization limit, the model can be solved exactly by treating H_V and H_W at the Hartree level [9]. The total entropy at chemical potential μ and inverse temperature β is given by

$$S_{\mathrm{Tot}}(\beta,\mu) = S_c(\beta,\mu) + S^{\mathrm{At}}(\beta,\mu). \tag{I7}$$

Here, $S^{\mathrm{At}}(\beta,\mu)$ is the entropy of the f-electrons as defined in Eq. (H9), while $S_c(\beta,\mu)$ represents the entropy of the c-electrons within the zero-hybridization model.

Let $\epsilon_{\mathbf{k},n}(\beta,\mu)$ (for $1 \leq n \leq 4\times 2\times 2 = 16$) denote the grand-canonical quasi-particle energies of the c-electrons for the n-th c-electron band at momentum $\mathbf{k}$. These energies are computed self-consistently for each μ by solving the zero-hybridization model following Ref. [11]. The explicit expression for the c-electron entropy then reads

$$S_c(\beta,\mu) = -\sum_{\mathbf{k},n}\left[n_F(\epsilon_{\mathbf{k},n}(\beta,\mu))\log(n_F(\epsilon_{\mathbf{k},n}(\beta,\mu))) + (1-n_F(\epsilon_{\mathbf{k},n}(\beta,\mu)))\log(1-n_F(\epsilon_{\mathbf{k},n}(\beta,\mu)))\right] \quad \text{(I8)}$$

where $n_F(\omega) = \frac{1}{e^{\beta\omega}+1}$ is the Fermi-Dirac distribution at inverse temperature β.

Note that our simplified model cannot reproduce the second entropy increase observed experimentally. Additional contribution, such as enhanced f-electron charge fluctuations, phonon contributions, or finite f-c hybridization, may account for this behavior but incorporating them would require a significantly more elaborate calculation, which lies beyond the scope of this work. Nevertheless, even though this issue is not central to our conclusions, we have added a figure showing the extended temperature range in Fig. S11. As seen in the plot, once the entropy reaches the second plateau, it increases only very slowly with temperature. This slow rise reflects the limited contribution from the c-electrons and from the strongly temperature-suppressed f-electron charge fluctuations within the zero-hybridization approximation. We stress, however, that simulations in this high-temperature regime are not strictly justified, since several additional entropy sources are ignored. For instance, even a small f–c hybridization would enhance the f-electron charge fluctuations and lead to a further entropy increase. Our omission of this extended-temperature simulation was therefore based on avoiding results obtained outside the regime of validity of the zero-hybridization approximation.

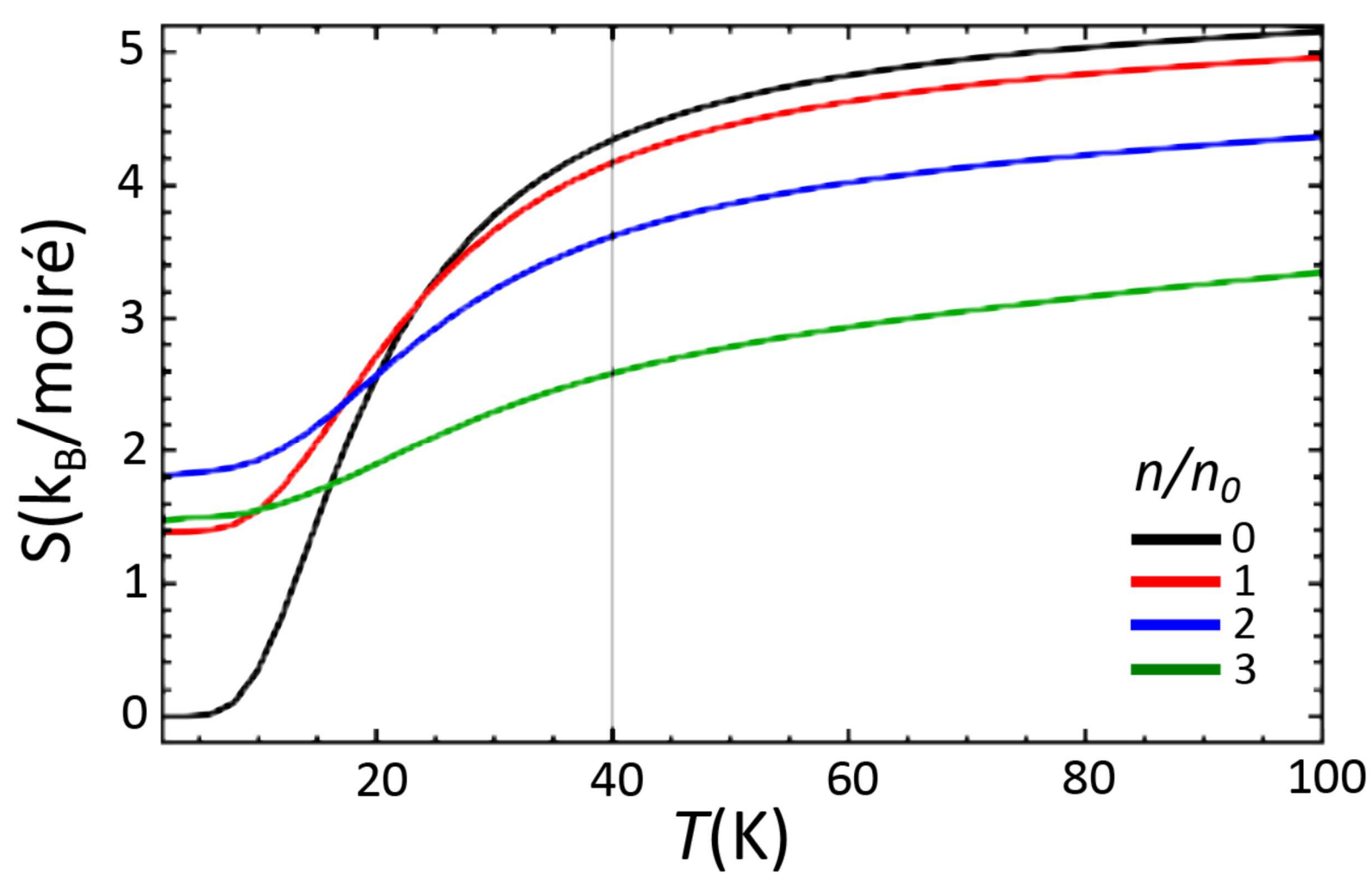

FIG. S11. **Simulation of entropy vs. temperature** Entropy at integer fillings calculated in the atomic limit of the topological heavy-fermion model with strain within broader temperature range from 2 K to 100K. The Hubbard interaction energy U_1 of 23 mV and the compressive heterostrain $\epsilon = 0.001$ are set to match the experimental data.

Appendix J: The phonon contribution to entropy

In this appendix, we consider the potential phonon effects on the entropy of TBG. We qualitatively estimate the entropy of phonons in TBG in the low-temperature limit.

The low-energy phonon spectrum of monolayer graphene comprises two in-plane linearly dispersing acoustic modes – the longitudinal acoustic (LA) and the transverse acoustic (TA) modes – and one out-of-plane quadratically dispersing mode, known as the z-directional acoustic (ZA) mode. The dispersion relations for these three modes are given by

$$\omega_{\mathrm{TA}}(\mathbf{k}) = v_{\mathrm{TA}}|\mathbf{k}|, \quad \omega_{\mathrm{LA}}(\mathbf{k}) = v_{\mathrm{LA}}|\mathbf{k}|, \quad \omega_{\mathrm{ZA}}(\mathbf{k}) = \frac{|\mathbf{k}|^2}{2m_{\mathrm{ZA}}}, \quad \text{(J1)}$$

where $v_{\mathrm{TA}} = 0.10\,\mathrm{eV\,Å}$, $v_{\mathrm{LA}} = 0.16\,\mathrm{eV\,Å}$, and $m_{\mathrm{ZA}} = 1.1\times 10^{-4} m_e$ (with m_e being the electron mass) [29, 30].

To estimate the low-temperature entropy of twisted bilayer graphene, we make the approximation of neglecting interlayer coupling. Additionally, we employ the Debye model for the phonon spectrum, which includes only acoustic phonons. The dispersion of the acoustic phonons is assumed to hold up to the edge of the Brillouin zone (BZ), which, for simplicity, is taken to be circular. The resulting "radius" of the BZ is known as the Debye wave vector K_D, chosen so that the circular BZ contains as many states as the original BZ. Letting N_{G} denote the number of graphene unit cells in one layer of the system and Ω the total surface area, we have

$$\frac{\pi K_D^2}{\frac{(2\pi)^2}{\Omega}} = N_{\mathrm{G}}, \tag{J2}$$

which implies

$$K_D = \sqrt{\frac{4\pi N_G}{\Omega}}. \tag{J3}$$

The internal phononic energy associated with the moiré unit cell (denoted by Ω_0) is given by

$$\begin{aligned}
U_{\mathrm{Phonon}}(\beta) &= 2\frac{\Omega_0}{\Omega}\int_0^\infty \mathrm{d}\omega \int_{|\mathbf{k}|\le K_D} \frac{\mathrm{d}^2 k}{\frac{(2\pi)^2}{\Omega}} \frac{\omega}{e^{\beta\omega}-1}\left(\delta\left(\omega_{\mathrm{TA}}(\mathbf{k})-\omega\right)+\delta\left(\omega_{\mathrm{LA}}(\mathbf{k})-\omega\right)+\delta\left(\omega_{\mathrm{ZA}}(\mathbf{k})-\omega\right)\right)\\
&= \frac{\Omega_0}{2\pi^2}\int_0^\infty \mathrm{d}\omega \int_0^{K_D} 2\pi k\,\mathrm{d}k\, \frac{\omega}{e^{\beta\omega}-1}\left(\frac{1}{v_{\mathrm{TA}}}\delta\left(k-\frac{\omega}{v_{\mathrm{TA}}}\right)+\frac{1}{v_{\mathrm{LA}}}\delta\left(k-\frac{\omega}{v_{\mathrm{LA}}}\right)+\frac{m_{\mathrm{ZA}}}{k}\delta\left(k-\sqrt{2m_{\mathrm{ZA}}\omega}\right)\right)\\
&= \frac{\Omega_0}{\pi}\int_0^\infty \mathrm{d}\omega\, \frac{\omega}{e^{\beta\omega}-1}\left(\frac{\omega}{v_{\mathrm{TA}}^2}\Theta\left(K_D-\frac{\omega}{v_{\mathrm{TA}}}\right)+\frac{\omega}{v_{\mathrm{LA}}^2}\Theta\left(K_D-\frac{\omega}{v_{\mathrm{LA}}}\right)+m_{\mathrm{ZA}}\Theta\left(K_D-\sqrt{2m_{\mathrm{ZA}}\omega}\right)\right)\\
&= \frac{\Omega_0}{\pi}\int_0^\infty \mathrm{d}x\, \frac{1}{e^x-1}\left(\frac{x^2}{\beta^3 v_{\mathrm{TA}}^2}\Theta\left(\beta v_{\mathrm{TA}}K_D-x\right)+\frac{x^2}{\beta^3 v_{\mathrm{LA}}^2}\Theta\left(\beta v_{\mathrm{LA}}K_D-x\right)+\frac{m_{\mathrm{ZA}}x}{\beta^2}\Theta\left(\frac{\beta K_D^2}{2m_{\mathrm{ZA}}}-x\right)\right)\\
&\approx \frac{\Omega_0}{\pi}\left[\frac{1}{\beta^3}\left(\frac{1}{v_{\mathrm{TA}}^2}+\frac{1}{v_{\mathrm{LA}}^2}\right)\int_0^\infty \mathrm{d}x\,\frac{x^2}{e^x-1}+\frac{m_{\mathrm{ZA}}}{\beta^2}\int_0^\infty \mathrm{d}x\,\frac{x}{e^x-1}\right]\\
&\approx \frac{\Omega_0}{\pi}\left[\frac{\Gamma(3)\zeta(3)}{\beta^3}\left(\frac{1}{v_{\mathrm{TA}}^2}+\frac{1}{v_{\mathrm{LA}}^2}\right)+\frac{m_{\mathrm{ZA}}\Gamma(2)\zeta(2)}{\beta^2}\right]\\
&\approx \frac{\Omega_0}{\pi}\left[\frac{2\zeta(3)}{\beta^3}\left(\frac{1}{v_{\mathrm{TA}}^2}+\frac{1}{v_{\mathrm{LA}}^2}\right)+\frac{\pi^2 m_{\mathrm{ZA}}}{6\beta^2}\right].
\end{aligned} \tag{J4}$$

The factor of two accounts for the two graphene layers in TBG. The internal energy was approximated in the low-temperature limit by assuming $\beta v_{\mathrm{TA}}K_D, \beta v_{\mathrm{LA}}K_D, \frac{\beta K_D^2}{2m_{\mathrm{ZA}}} \gg 1$. In Eq. (J4), $\zeta(n)$ denotes the Riemann zeta function. The corresponding specific heat is given by

$$C_{\mathrm{Phonon}}(\beta) = \frac{\mathrm{d}U_{\mathrm{Phonon}}(\beta)}{\mathrm{d}\left(\frac{1}{\beta}\right)} \approx \frac{\Omega_0}{\pi}\left[\frac{6\zeta(3)}{\beta^2}\left(\frac{1}{v_{\mathrm{TA}}^2}+\frac{1}{v_{\mathrm{LA}}^2}\right)+\frac{\pi^2 m_{\mathrm{ZA}}}{3\beta}\right]. \tag{J5}$$

Consequently, the phonon contribution to the entropy of graphene is

$$\begin{aligned}
S_{\mathrm{Phonon}}(\beta) &= \int_0^\beta \beta C_{\mathrm{Phonon}}(\beta)\,\mathrm{d}\left(\frac{1}{\beta}\right) \approx \frac{\Omega_0}{\pi}\left[\frac{6\zeta(3)}{\beta^2}\left(\frac{1}{v_{\mathrm{TA}}^2}+\frac{1}{v_{\mathrm{LA}}^2}\right)+\frac{\pi^2 m_{\mathrm{ZA}}}{3\beta}\right]\\
&\approx \frac{\Omega_0}{\pi}\left[\frac{3\zeta(3)}{\beta^2}\left(\frac{1}{v_{\mathrm{TA}}^2}+\frac{1}{v_{\mathrm{LA}}^2}\right)+\frac{\pi^2 m_{\mathrm{ZA}}}{3\beta}\right]\\
&\approx \left(\frac{\beta_{\mathrm{TA/LA}}}{\beta}\right)^2+\left(\frac{\beta_{\mathrm{ZA}}}{\beta}\right).
\end{aligned} \tag{J6}$$

In Eq. (J6), we have introduced the characteristic inverse temperatures for the acoustic modes of graphene

$$\begin{aligned}
\frac{1}{\beta_{\mathrm{TA/LA}}} &= \left[\frac{3\zeta(3)\Omega_0}{\pi}\left(\frac{1}{v_{\mathrm{TA}}^2}+\frac{1}{v_{\mathrm{LA}}^2}\right)\right]^{-\frac{1}{2}} &&\approx 7\,\mathrm{K},\\
\frac{1}{\beta_{\mathrm{ZA}}} &= \left(\frac{\pi\Omega_0 m_{\mathrm{ZA}}}{3}\right)^{-1} &&\approx 5\times 10^4\,\mathrm{K}.
\end{aligned} \tag{J7}$$

The estimation in Eq. (J6) indicates that the in-plane acoustic modes of graphene begin to contribute significantly to the entropy of TBG around 7 K. In the absence of electron-phonon coupling, this phonon contribution would not affect the experimentally measured entropy, as the experiment measures the difference between the entropy of TBG at a given filling ν and at the band edge.

However, due to electron-phonon coupling effects, the acoustic phonon dispersion can acquire a weak dependence on the filling, leading to phonon contributions in the experimentally measured entropy. Since the acoustic phonon contribution becomes relevant around 7 K and subsequently grows quadratically with temperature, this effect can account for the observed increase in entropy beyond the second plateau at temperatures much lower than the f-electron interaction energy scale or the characteristic energy dispersion of the c-electrons.

---

[1] M. A. Altvater, S. Wu, Z. Zhang, T. Zhu, G. Li, K. Watanabe, T. Taniguchi, and E. Y. Andrei, 2D Materials **6**, 045034 (2019).

[2] L. Britnell, R. V. Gorbachev, R. Jalil, B. D. Belle, F. Schedin, A. Mishchenko, T. Georgiou, M. I. Katsnelson, L. Eaves, S. V. Morozov, N. M. R. Peres, J. Leist, A. K. Geim, K. S. Novoselov, and L. A. Ponomarenko, Science **335**, 947 (2012), https://www.science.org/doi/pdf/10.1126/science.1218461.

[3] K. Shiga, T. Komiyama, Y. Fuse, H. Fukidome, A. Sato, T. Otsuji, and T. Uchino, Japanese Journal of Applied Physics **59**, SIID03 (2020).

[4] H. Yang, J. Heo, S. Park, H. J. Song, D. H. Seo, K.-E. Byun, P. Kim, I. Yoo, H.-J. Chung, and K. Kim, Science **336**, 1140 (2012), https://www.science.org/doi/pdf/10.1126/science.1220527.

[5] Z.-D. Song and B. A. Bernevig, Phys. Rev. Lett. **129**, 047601 (2022).

[6] L. L. H. Lau and P. Coleman, arXiv:2303.02670 [cond-mat] 10.48550/arXiv.2303.02670 (2023), arXiv:2303.02670 [cond-mat].

[7] D. Călugăru, M. Borovkov, L. L. H. Lau, P. Coleman, Z.-D. Song, and B. A. Bernevig, Low Temp. Phys. **49**, 640 (2023).

[8] J. Yu, M. Xie, B. A. Bernevig, and S. Das Sarma, Phys. Rev. B **108**, 035129 (2023).

[9] H. Hu, B. A. Bernevig, and A. M. Tsvelik, Phys. Rev. Lett. **131**, 026502 (2023).

[10] Y.-Z. Chou and S. Das Sarma, Phys. Rev. Lett. **131**, 026501 (2023).

[11] H. Hu, G. Rai, L. Crippa, J. Herzog-Arbeitman, D. Călugăru, T. Wehling, G. Sangiovanni, R. Valentí, A. M. Tsvelik, and B. A. Bernevig, Phys. Rev. Lett. **131**, 166501 (2023).

[12] G.-D. Zhou, Y.-J. Wang, N. Tong, and Z.-D. Song, Phys. Rev. B **109**, 045419 (2024).

[13] R. L. Merino, D. Calugaru, H. Hu, J. Diez-Merida, A. Diez-Carlon, T. Taniguchi, K. Watanabe, P. Seifert, B. A. Bernevig, and D. K. Efetov, arXiv:2402.11749 [cond-mat] 10.48550/arXiv.2402.11749 (2024), arXiv:2402.11749 [cond-mat].

[14] S. Batlle-Porro, D. Calugaru, H. Hu, R. K. Kumar, N. C. H. Hesp, K. Watanabe, T. Taniguchi, B. A. Bernevig, P. Stepanov, and F. H. L. Koppens, arXiv:2402.00869 [cond-mat] 10.48550/arXiv.2402.12296 (2024), arXiv:2402.00869 [cond-mat].

[15] Y.-J. Wang, G.-D. Zhou, S.-Y. Peng, B. Lian, and Z.-D. Song, arXiv:2402.00869 [cond-mat] 10.48550/arXiv.2402.00869 (2024), arXiv:2402.00869 [cond-mat].

[16] D. Călugăru, H. Hu, R. L. Merino, N. Regnault, D. K. Efetov, and B. A. Bernevig, arXiv:2402.14057 [cond-mat] (2024), arXiv:2402.14057 [cond-mat].

[17] G. Rai, L. Crippa, D. Călugăru, H. Hu, F. Paoletti, L. de' Medici, A. Georges, B. A. Bernevig, R. Valentí, G. Sangiovanni, and T. Wehling, Phys. Rev. X **14**, 031045 (2024).

[18] J. Herzog-Arbeitman, J. Yu, D. Călugăru, H. Hu, N. Regnault, O. Vafek, J. Kang, and B. A. Bernevig, arXiv:2405.13880 [cond-mat] 10.48550/arXiv.2405.13880 (2024), arXiv:2405.13880 [cond-mat].

[19] J. Herzog-Arbeitman, D. Călugăru, H. Hu, J. Yu, N. Regnault, J. Kang, B. A. Bernevig, and O. Vafek, arXiv:2502.08700 [cond-mat] 10.48550/arXiv.2502.08700 (2025), arXiv:2502.08700 [cond-mat].

[20] H. Hu, Z.-D. Song, and B. A. Bernevig, arXiv:2502.14039 [cond-mat] 10.48550/arXiv.2502.14039 (2025), arXiv:2502.14039 [cond-mat].

[21] D. Călugăru, N. Regnault, M. Oh, K. P. Nuckolls, D. Wong, R. L. Lee, A. Yazdani, O. Vafek, and B. A. Bernevig, Phys. Rev. Lett. **129**, 117602 (2022).

[22] Y. H. Kwan, G. Wagner, T. Soejima, M. P. Zaletel, S. H. Simon, S. A. Parameswaran, and N. Bultinck, Phys. Rev. X **11**, 041063 (2021).

[23] P. Pulay, Chemical Physics Letters **73**, 393 (1980).

[24] P. Pulay, Journal of Computational Chemistry **3**, 556 (1982).

[25] E. Cancès and C. Le Bris, International Journal of Quantum Chemistry **79**, 82 (2000).

[26] K. N. Kudin, G. E. Scuseria, and E. Cancès, The Journal of Chemical Physics **116**, 8255 (2002).

[27] A. J. Garza and G. E. Scuseria, The Journal of Chemical Physics **137**, 054110 (2012).

[28] D. Călugăru and others, To be published (2024).

[29] X. Cong, Q.-Q. Li, X. Zhang, M.-L. Lin, J.-B. Wu, X.-L. Liu, P. Venezuela, and P.-H. Tan, Carbon **149**, 19 (2019).

[30] A. A. Balandin, ACS Nano **14**, 5170 (2020).
