# Supplementary material for: Entropy plateaus, combinatorial degeneracy, local moments, and heavy-fermion mass renormalization in magic-angle graphene
Source: Nat Commun. 2026 Aug 8;17:9785. doi: 10.1038/s41467-026-76179-y (PMC13575161; doi:10.1038/s41467-026-76179-y)
Supplement: Supplementary file 2 [file 41467_2026_76179_MOESM2_ESM.pdf]

nature portfolio

Peer Review File

Local Moment Formation, Entropy Plateaus, Combinatorial Degeneracy, and Heavy-Fermion Mass Renormalization in Magic-Angle Graphene

Corresponding Author: Professor Eva Andrei

This file contains all reviewer reports in order by version, followed by all author rebuttals in order by version.

This manuscript has been previously reviewed at another journal. This document only contains information relating to versions considered at Nature Communications.

Version 0:

Reviewer comments:

Reviewer #2

(Remarks to the Author)
The authors have revised the manuscript and provided detailed responses to the referee comments. The revised version has improved in clarity, and several concerns have been addressed.

In particular, the authors have clarified the distinctions between their planar tunneling spectroscopy (PTS) approach and prior scanning SET-based measurements. The discussion on entropy extraction, temperature calibration, and effective mass interpretation has also been expanded and is now more transparent. These additions help strengthen the technical foundation of the work.

Regarding the concern about novelty relative to prior studies, while it remains true that several individual observables (e.g., compressibility cascades, entropy extraction, and mass renormalization) have been reported in earlier works, the present manuscript combines these measurements within a unified experimental platform.

That said, some of the broader claims regarding heavy-fermion physics and its implications may still rely partly on interpretation rather than entirely unambiguous experimental signatures. However, the combination of methodological advance and the reported phenomenology is sufficient to justify publication.

Overall, I believe the manuscript, in its revised form, meets the standard for publication in Nature Communications.

Reviewer #3

(Remarks to the Author)
We have considered all comments and responses from the previous round. While we are convinced by many of the answers, there are still a few remaining points we would like to ask the authors to clarify:

In the answer to our point 3, the authors write “We have updated Fig. 4d to show the entropy over a broader temperature range, making the second plateau more clearly visible.”. Maybe we are missing something or obtained the wrong version of the manuscript, however, the updated entropy plot – now Fig. 2e – seems to be unchanged. As such, we still do not really see plateaus.

Apart from this, we found the following minor typos:

There is a typo on page 10. “(SI Appendix I)” is written twice: The absence of further increase at higher temperatures may be due to the omission of additional phonon contributions or f-c hybridization effects (SI Appendix I). (SI Appendix I). [21, 37].

There is a typo on page 14, “ccc-like”: Within this framework, hole-like excitations near integer fillings acquire predominantly f-electron character, while electron-like excitations remain largely ccc-like.

Reviewer #4

(Remarks to the Author)
I co-reviewed this manuscript with one of the reviewers who provided the listed reports. This is part of the Nature Communications initiative to facilitate training in peer review and to provide appropriate recognition for Early Career Researchers who co-review manuscripts.

Version 1:

Reviewer comments:

Reviewer #3

(Remarks to the Author)
I am OK with the current version of the manuscript.

Reviewer #4

(Remarks to the Author)
I co-reviewed this manuscript with one of the reviewers who provided the listed reports. This is part of the Nature Communications initiative to facilitate training in peer review and to provide appropriate recognition for Early Career Researchers who co-review manuscripts.

Open Access This Peer Review File is licensed under a Creative Commons Attribution 4.0 International License, which permits use, sharing, adaptation, distribution and reproduction in any medium or format, as long as you give appropriate credit to the original author(s) and the source, provide a link to the Creative Commons license, and indicate if changes were made.
In cases where reviewers are anonymous, credit should be given to 'Anonymous Referee' and the source.
The images or other third party material in this Peer Review File are included in the article's Creative Commons license, unless indicated otherwise in a credit line to the material. If material is not included in the article's Creative Commons license and your intended use is not permitted by statutory regulation or exceeds the permitted use, you will need to obtain permission directly from the copyright holder.
To view a copy of this license, visit https://creativecommons.org/licenses/by/4.0/

Reponses to Referees

Referee #3:

In the answer to our point 3, the authors write “We have updated Fig. 4d to show the entropy over a broader temperature range, making the second plateau more clearly visible.”. Maybe we are missing something or obtained the wrong version of the manuscript, however, the updated entropy plot – now Fig. 2e – seems to be unchanged. As such, we still do not really see plateaus

Response: We have already provided our reply in the second round of revisions; however, it appears that the reviewer may not have had the opportunity to see it. We therefore restate our response to the concerns below for clarity. We thank the referee for raising this point. Before addressing this specific comment, we would like to reiterate the strengths and limitations of the zero-hybridization model. The first entropy plateau associated with the four-fold–degenerate *f*-electron local-moment states is clearly visible in Fig. 2e. However, as we have emphasized throughout the manuscript, the zero-hybridization approximation necessarily omits several key physical ingredients required for a fully quantitative description of the entropy at higher temperatures. For this reason, we are not certain about the motivation for extending the temperature range, as the model we employ does not capture any entropy increase beyond the second plateau.

Note that our simplified model cannot reproduce the second entropy increase observed experimentally. Additional contribution, such as enhanced *f*-electron charge fluctuations, phonon contributions, or finite *f*-*c* hybridization, may account for this behavior but incorporating them would require a significantly more elaborate calculation, which lies beyond the scope of this work. Nevertheless, even though this issue is not central to our conclusions, we have added a figure showing the extended temperature range in the Appendix I of the Supplementary Information (see Figure R1 below). As seen in the plot, once the entropy reaches the second plateau, it increases only very slowly with temperature. This slow rise reflects the limited contribution from the *c*-electrons and from the strongly temperature-suppressed *f*-electron charge

fluctuations within the zero-hybridization approximation. We stress, however, that simulations in this high-temperature regime are not strictly justified, since several additional entropy sources are ignored. For instance, even a small *f*–*c* hybridization would enhance the *f*-electron charge fluctuations and lead to a further entropy increase. A rough estimate of the temperature at which phonon contributions to entropy are expected to play a significant role is provided in Appendix J of the Supplementary Information. Our omission of this extended-temperature simulation was therefore based on avoiding results obtained outside the regime of validity of the zero-hybridization approximation. The related discussions are added in Appendix I of the Supplementary Information.

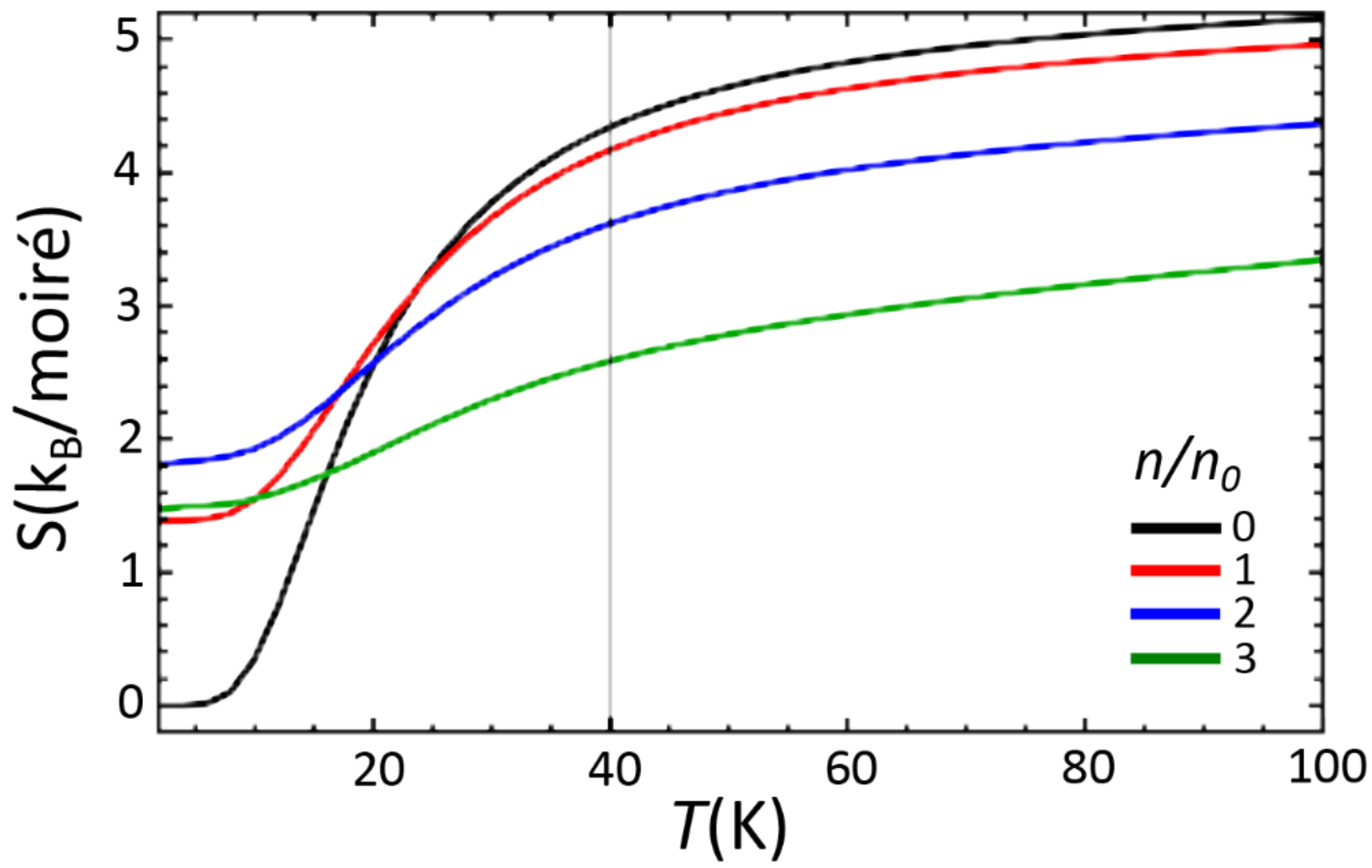

Figure R1 Entropy at integer fillings calculated in the atomic limit of the topological heavy-fermion model with strain within broader temperature range from 2K to 100K. The Hubbard interaction energy U_1 of 23mV and the compressive heterostrain ε=0.001 are set to match the experimental data.

There is a typo on page 10. "(SI Appendix I)" is written twice: The absence of further increase at higher temperatures may be due to the omission of additional phonon contributions or f-c hybridization effects (SI Appendix I). (SI Appendix I). [21, 37].

Response: The typo is fixed.

There is a typo on page 14, “ccc-like”: Within this framework, hole-like excitations near integer fillings acquire predominantly f-electron character, while electron-like excitations remain largely ccc-like.

Response: The typo is fixed.
